# Recognition of Streptococcal Promoters by the Pneumococcal SigA Protein

**DOI:** 10.3389/fmolb.2021.666504

**Published:** 2021-06-24

**Authors:** Virtu Solano-Collado, Sofía Ruiz-Cruz, Fabián Lorenzo-Díaz, Radoslaw Pluta, Manuel Espinosa, Alicia Bravo

**Affiliations:** ^1^ Institute of Medical Sciences, University of Aberdeen, Aberdeen, United Kingdom; ^2^ School of Microbiology, University College Cork, Cork, Ireland; ^3^ Departamento de Bioquímica, Microbiología, Biología Celular y Genética, Universidad de La Laguna, Tenerife, Spain; ^4^ Institute for Research in Biomedicine (IRB Barcelona), The Barcelona Institute of Science and Technology, Barcelona, Spain; ^5^ Centro de Investigaciones Biológicas Margarita Salas, Consejo Superior de Investigaciones Científicas, Madrid, Spain

**Keywords:** plasmid pMV158, RNA polymerase, SigA protein, sigma factor, streptococcal promoters, *Streptococcus pneumoniae*

## Abstract

Promoter recognition by RNA polymerase is a key step in the regulation of gene expression. The bacterial RNA polymerase core enzyme is a complex of five subunits that interacts transitory with one of a set of sigma factors forming the RNA polymerase holoenzyme. The sigma factor confers promoter specificity to the RNA polymerase. In the Gram-positive pathogenic bacterium *Streptococcus pneumoniae*, most promoters are likely recognized by SigA, a poorly studied housekeeping sigma factor. Here we present a sequence conservation analysis and show that SigA has similar protein architecture to *Escherichia coli* and *Bacillus subtilis* homologs, namely the poorly conserved N-terminal 100 residues and well-conserved rest of the protein (domains 2, 3, and 4). Further, we have purified the native (untagged) SigA protein encoded by the pneumococcal R6 strain and reconstituted an RNA polymerase holoenzyme composed of the *E. coli* core enzyme and the sigma factor SigA (RNAP-SigA). By *in vitro* transcription, we have found that RNAP-SigA was able to recognize particular promoters, not only from the pneumococcal chromosome but also from the *S. agalactiae* promiscuous antibiotic-resistance plasmid pMV158. Specifically, SigA was able to direct the RNA polymerase to transcribe genes involved in replication and conjugative mobilization of plasmid pMV158. Our results point to the versatility of SigA in promoter recognition and its contribution to the promiscuity of plasmid pMV158.

## Introduction

Bacteria in their natural habitats are often subjected to rapid changes in the provision of nutrients (the so-called feast-famine situation). These environmental fluctuations may lead from moderate to drastic changes in the bacterial lifestyle, affecting many transcription regulatory pathways of the bacterial cells (Yokoyama et al., 2006). Transcription patterns can also be severely affected when bacteria colonize new niches where the availability of nutrients can be far different (Bravo et al., 2018). This is true in the case of the Gram-positive bacterium *Streptococcus pneumoniae* (the pneumococcus), which colonizes the human nasopharynx of about 70% of healthy individuals (Gamez and Hammerschmidt, 2012). However, under hospital conditions or in immunocompromised individuals, pneumococci can invade, among other niches, the lower respiratory tract, thus leading to pneumococcal pneumonia. This deadly infection causes up to 1.5 million deaths per year (Dockrell et al., 2012). Pneumococcal pneumonia is the single largest infectious cause of death in children worldwide, being responsible for the death of nearly 1 million children under the age of five in 2017 (https://www.who.int/en/news-room/fact-sheets/detail/pneumonia). Also, pneumococcal pneumonia is a heavy economic burden on society (O’Brien et al., 2009). Changes in the pneumococcal transcriptome under various infection-relevant conditions have been reported, revealing profound changes in the relative amount of the RNAs synthesized by this microorganism (Aprianto et al., 2018).

Transcription of bacterial genes requires the assembly of five protein subunits (α_2_ββ´ω) that constitute the RNA polymerase core enzyme. Subsequently, the core enzyme associates temporarily with one of a set of sigma factors forming the RNA polymerase holoenzyme. The holoenzyme, but not the core enzyme, can recognize promoters and initiate transcription. Promoter recognition by RNA polymerase is a crucial step in the regulation of gene expression. The promoter specificity of the RNA polymerase depends on its sigma factor, which can contact several sequence motifs, including the −35 element, the extended −10 element, the −10 element and the discriminator region (Browning and Busby, 2016).

Nearly all bacteria have various sigma factors that direct the RNA polymerase to transcribe particular genes depending on the bacterial needs. Nevertheless, it is the housekeeping sigma factor the one responsible for recognizing the majority of promoters and, consequently, for cell proliferation and wellbeing. Most of the transcription processes taking place in exponentially growing bacteria are initiated by the RNA polymerase that contains the housekeeping sigma factor (Saecker et al., 2011). Leading investigations on *Escherichia coli* identified the consensus sequences of the −35 (5′-TTGACA-3′) and −10 (5′-TATAAT-3′) elements recognized by its housekeeping sigma factor (σ70; also termed RpoD). The optimal spacer length between the two promoter elements was reported to be 17 nucleotides (nt). Compared to the housekeeping sigma factor, alternative sigma factors usually recognize a smaller set of promoters, have more stringent sequence specificities, and associate with fewer transcription factors (Browning and Busby, 2016). Moreover, numerous alternative sigma factors play key roles when bacteria enter into the stationary phase and/or they are confronted with stressful environmental conditions (Saecker et al., 2011). The housekeeping sigma factors have four main regions: 1.1, 2 (consisting of 1.2, 2.1–2.4), 3 (3.0–3.2) and 4 (4.1–4.2) that group into four structured helical domains (1.1, 2, 3, and 4). The poorly conserved domain 1.1 is found only in Group 1 of housekeeping sigma factors and is involved in restraining the sigma factor apo-form from DNA binding, thereby inhibiting its non-productive interaction with promoter DNA in the absence of the RNA polymerase core complex. In the RNA polymerase holoenzyme, the negatively charged domain 1.1 (a mimicry of DNA negative charge) needs to be displaced from its initial position for the DNA-RNA polymerase complex to become transcriptionally competent. The displacement process takes place more easily at some promoters while behaving more inhibitory or difficult to move out at others, therefore it assists in the promoter selection process (Paget, 2015; Fang et al., 2020; Shin et al., 2021).

In *S. pneumoniae*, most housekeeping genes are transcribed by RNA polymerase complexes that contain the sigma factor SigA, also known as RpoD and σ43. However, it is the alternative sigma factor SigX the best characterized so far (Luo et al., 2003; Inniss et al., 2019). SigA (369 amino acids) belongs to the family of σ70 factors, despite its small size, and is thought to be responsible for the transcription of most pneumococcal genes. SigX, the only known alternative sigma factor in pneumococcus (Inniss and Morrison, 2020), is involved in the transcription of a set of genes that participate in the development of genetic competence for transformation and is encoded by the *comX* gene (Tovpeko and Morrison, 2014). The pneumococcal promoters recognized by SigA share many features with those recognized by the σ70 factor of *E. coli* in terms of the consensus sequences defined for the −35 and −10 regions (Puyet and Espinosa, 1993; Sabelnikov et al., 1995; Ruiz-Cruz et al., 2010).

Although bioinformatics can predict promoter sequences in genomic DNAs, their definitive identification requires the use of different experimental approaches, both *in vivo* and *in vitro* (Ross and Gourse, 2009). This is particularly necessary when dealing with genomic DNAs that have a high A + T content, as it is the case of the pneumococcal R6 chromosome (60% A + T) (Hoskins et al., 2001) and the *S. agalactiae* plasmid pMV158 (63% A + T), which can replicate in a broad variety of bacterial species, including *S pneumoniae* and *E. coli* (Lacks et al., 1986; del Solar et al., 1987). In such DNAs, stretches resembling the consensus −10 element (5′-TATAAT-3′) of the promoters recognized by housekeeping sigma factors are frequent.

Bacterial RNA polymerase holoenzymes share a conserved architecture of the core enzyme (Griesenbeck et al., 2017). In the present work, we report the purification of the native (untagged version) pneumococcal SigA protein encoded by the strain R6, the reconstitution of RNA polymerase complexes constituted by the *E. coli* RNA polymerase core enzyme and the pneumococcal SigA factor (RNAP-SigA), and the ability of the RNAP-SigA complexes to recognize particular streptococcal promoters and initiate transcription. Specifically, we used DNA templates that contain promoters identified previously by *in vivo* approaches: i) the promoter region of the pneumococcal *mgaSpn* gene, which encodes a transcriptional regulator, and ii) the promoter regions of the *copG* (replication control) and *mobM* (conjugative mobilization) genes from the streptococcal plasmid pMV158. Our results support the contribution of SigA to the promiscuous behaviour of pMV158.

## Materials and Methods

### Bacterial Strains, Plasmids, and Oligonucleotides

*E. coli* BL21 (DE3) (a gift of F. W. Studier) was used for the overproduction of SigA. The strain is based on the DE3 lambda lysogen developed in Studier’s laboratory (Studier and Moffatt, 1986; Studier et al., 1990) in which the gene that encodes the RNA polymerase of bacteriophage T7 is under the control of the *lacUV5* promoter, inducible by isopropyl β-D-1-thiogalactopyranoside (IPTG). This bacterial strain is also deficient in the Lon protease. The pneumococcal *sigA* gene was cloned into the *E. coli* expression vector pET24b (Novagen), under the control of the ϕ10-promoter of phage T7, generating its derivative pET24b-*sigA* (see below). Both plasmid pET24b and the BL21 (DE3) chromosome carry the *lacI* repressor gene to ensure tight repression of the *lacUV5* promoter. For small-scale preparations of plasmid DNA, a High Pure Plasmid Isolation Kit (Roche Applied Science) was used. Chromosomal DNA was isolated from *S. pneumoniae* R6 (Hoskins et al., 2001) as previously described (Lacks, 1966). Plasmid pMV158 (Lacks et al., 1986; Priebe and Lacks, 1989) was purified from *S. pneumoniae* 708 (*trt*-1 *hex*-4 *end*-1 *exo*-2 *malM*594) (Lacks and Greenberg, 1977) by two consecutive CsCl gradients as previously described (del Solar et al., 1987). The oligonucleotides used in this work are listed in Table 1.

**TABLE 1:** Oligonucleotides used in this work.

Name	Sequence (5′–3′)^a^	Reference
*sigA*-F	GAA​GAA​TGG​AGT​A**CAT** **ATG**​GCA​ACA​AA	This work
*sigA*-R	TTC​AAT​TTG​CT**CTCGAG**TAT​AAG​CCA​T	This work
1622C	GAT​TCT​GTA​TTC​ACG​CCC​TC	Solano-Collado et al. (2013)
1622D	TTC​TAA​TTG​CCT​ATG​ACT​TTT​TTT​AG	Solano-Collado et al. (2013)
1622F	CGA​TGA​AAC​CAA​CGT​TTA​TGT​TC	Solano-Collado et al. (2013)
*copG-*F	CGC​CTT​TAG​CCT​TAG​AGC​TG	This work
*copG-*R	GAT​AAC​CCC​ATC​TCT​CTT​GCC	This work
*mobM-*F	GAG​GTG​GCA​GAG​GGC​AGG​TT	This work
*mobM-*R1	GCA​ACC​ATG​TAA​CTC​ATA​GA	This work
*mobM-*R2	GCT​TTC​ATC​TTC​TGC​ATT​CT	This work
1622A	AGT​TCC​TGA​TTG​TAT​TCC​CT	Solano-Collado et al. (2013)
1622B	CAC​AAC​ACT​GCC​TAC​CCT​CC	Solano-Collado et al. (2013)
1622H	CGG​ATT​AAA​CCT​CTT​GCA​ATT​ATA​CC	Solano-Collado et al. (2013)
1622I	CAA​ATT​CTT​TAA​TTG​TTG​CTA​TTA	Solano-Collado et al. (2013)
-40 M13	GTTTTCCCAGTCACGAC	Yanisch-Perron et al. (1985)
oligo-2	TCAGCATAACTGAGCC	Monti et al. (2007)
pr14686	CCC​AAA​AAG​TAG​CTT​CAC​TGC​G	Li et al. (2020)
pr14687	TAG​AAA​CTA​CTC​GAA​TTT​ATC​TAA​GGA​AAA​C	Li et al. (2020)
pr15159	CAT​AAG​ATA​GGA​GTT​TTC​ATA​TGA​AAG​ATT​TTG	Li et al. (2020)

a Restriction sites are in bold. Bases changed to generate restriction sites are underlined.

### Growth and Transformation of Bacteria

*E. coli* BL21 (DE3) cells harbouring a pET24b derivative were grown in tryptone-yeast extract (TY) medium (Pronadisa) supplemented with kanamycin (Km; 30 μg/ml), at 37°C under aerobic conditions. Bacterial growth was measured by determination of the optical density of the cultures at 600 nm (OD_600_). The protocol used to transform *E. coli* by electroporation has been described (Dower et al., 1988). *S. pneumoniae* cells were grown in AGCH medium (Lacks, 1968; Ruiz-Cruz et al., 2010) supplemented with 0.3% sucrose and 0.2% yeast extract, at 37°C in a static water bath. Bacterial growth was measured at OD_650_. For pneumococcal cells harbouring plasmid pMV158, the medium was supplemented with tetracycline (1 µg/ml).

### Polymerase Chain Reaction

The Phusion High-Fidelity DNA polymerase (Thermo Scientific) and the Phusion HF buffer were used. Reaction mixtures (50 µl) contained 5–30 ng of template DNA, 20 pmol of each primer, 200 µM each dNTP, and one unit of DNA polymerase. PCR conditions have been described (Ruiz-Cruz et al., 2010). PCR products were purified with the QIAquick PCR purification kit (QIAGEN).

### Construction of Plasmid pET24b-*sigA*

The complete genome sequence of *S. pneumoniae* R6 has been published (Hoskins et al., 2001) (NCBI RefSeq NC_003098.1). The *sigA* gene (locus_tag SPR_RS04905; coordinates 965,281–966,390) encodes the sigma factor SigA (also known as RpoD). To overproduce SigA, a 1,166-bp DNA region of the pneumococcal R6 chromosome, which contains the promoter-less *sigA* gene was amplified by PCR using the *sigA*-F and *sigA*-R oligonucleotides (Table 1). These primers have a single restriction site for *Nde*I and *Xho*I, respectively. The amplified product was digested with both enzymes, and the 1,142-bp restriction fragment was ligated to pET24b digested with the same enzymes. *E. coli* BL21 (DE3) transformants harbouring the recombinant plasmid (pET24b-*sigA*) were selected, and the inserted fragment and the regions of pET24b flanking the inserted fragment were sequenced. Dye-terminator sequencing was carried out at Secugen (CIB Margarita Salas, Madrid, Spain).

### Overproduction and Purification of SigA

*E. coli* BL21 (DE3) cells carrying plasmid pET24b-*sigA* were grown at 37°C with rotary shaking in TY medium containing Km (30 µg/ml) to an OD_600_ of 0.45. Then, IPTG (1 mM) was added to induce the expression of the T7 RNA polymerase-encoding gene and, therefore, the expression of *sigA*. After 25 min, rifampicin (200 µg/ml) was added to inhibit specifically the activity of the *E. coli* RNA polymerase. After 60 min, cells were harvested by centrifugation (9,000 rpm in an SLA-3000 rotor for 20 min at 4°C) and washed twice with buffer A (50 mM Tris-HCl, pH 7.6, 5% glycerol, 1 mM DTT, 1 mM EDTA) containing 400 mM NaCl. The cell pellet was concentrated (40x) in buffer A containing 400 mM NaCl and a protease inhibitor cocktail (Roche). Cells were disrupted by two passages through a pre-chilled French pressure cell, and the whole-cell extract was centrifuged (10,000 rpm in an Eppendorf F-34-6-38 rotor for 40 min at 4°C). The cleared lysate was mixed with 0.2% polyethyleneimine (PEI), kept on ice for 30 min, and centrifuged (9,000 rpm in an Eppendorf F-34-6-38 rotor for 20 min at 4°C). Under these conditions, SigA was recovered in the PEI pellet, which was then washed with buffer A containing 100 mM NaCl. SigA was eluted from the PEI pellet with buffer A containing 700 mM NaCl. Proteins recovered in the supernatant were precipitated with 70% saturated ammonium sulphate. After centrifugation (9,000 rpm in an Eppendorf F-34-6-38 rotor for 20 min at 4°C), the precipitate was dissolved in buffer A containing 100 mM NaCl and dialyzed against the same buffer at 4°C. The protein preparation was applied to a heparin affinity column (Affi-gel BioRad Heparin Gel, Econo-column BioRad) equilibrated with buffer A containing 100 mM NaCl. About 10% of SigA was in the flow-through, and about 80% of SigA was recovered when the column was washed with buffer A containing 100 mM NaCl. The latter protein fraction was concentrated by filtering through a 10-kDa-cutoff membrane (Macrosep, Pall), and dialyzed against buffer A containing 100 mM NaCl. Then, gel filtration chromatography was carried out in an AKTA HPLC system (Amersham Biosciences) using a HiLoad Superdex 200 gel filtration column (Amersham Biosciences), equilibrated with buffer A containing 100 mM NaCl. Fractions were analysed by Coomassie-stained SDS polyacrylamide (10%) gels, pooled, concentrated as described above, and stored at −80°C.

### DNA Regions Amplified by Polymerase Chain Reaction

The following DNA regions were amplified by PCR: a) a 224-bp region of the R6 chromosome (coordinates 1,598,452–1,598,229) using the 1622C and 1622D oligonucleotides, b) a 265-bp region of the R6 chromosome (coordinates 1,598,452–1,598,188) using the 1622C and 1622F oligonucleotides, c) a 246-bp region of the pMV158 plasmid (NCBI RefSeq NC_010096.1; coordinates 489–734) using the *copG*-F and *copG*-R oligonucleotides, d) a 288-bp region of the pMV158 plasmid (coordinates 3,461–3,748) using the *mobM*-F and *mobM*-R1 oligonucleotides, and e) a 309-bp region of the pMV158 plasmid (coordinates 3,461–3,769) using the *mobM*-F and *mobM*-R2 oligonucleotides. The oligonucleotides are listed in Table 1.

### *In vitro* Transcription Assays

The interaction of purified RNA polymerase with a particular promoter is affected greatly by several parameters, including temperature (Ross and Gourse, 2009). Although the optimum conditions for RNA polymerase complex formation with a particular promoter must be determined empirically, typical multiple-round *in vitro* transcription reactions are incubated at 25, 30 or 37°C (Ross and Gourse, 2009). Linear DNA fragments amplified by PCR were used as templates for *in vitro* transcription assays under multiple-round conditions. The procedure involved three steps. First, the reaction mixture (41.5 µl) contained 0.5 µl DTT (100 mM), 0.5 µl BSA (10 mg/ml), 10 µl transcription buffer (200 mM Tris-HCl, pH 7.5, 750 mM KCl, 50 mM MgCl_2_, 0.05% Triton X-100), 1 µl (700 ng; 1 unit) *E. coli* RNA polymerase core (Epicentre), 0.5 µl (250 ng) SigA and 29 µl H_2_O. The reaction mixture was incubated at 30°C for 15 min (*Reconstitution Step*). Then, 2.5 µl of DNA template (200 nM) was added and the reaction mixture was incubated at 30°C for 15 min (*Binding Step*). Finally, 0.5 µl (20 units) SUPERase-In (RNase inhibitor, Ambion), 5 µl rNTPs (2.5 mM of each rNTP) and 0.5 µl (α-^32^P)-UTP (10 µCi/µl; 3,000 Ci/mmol; GE Healthcare) were added to the reaction mixture, which was then incubated at 30°C for 15 min (*Transcription Step*). As a control, transcription reactions using *E. coli* RNA polymerase holoenzyme (500 ng/µl; 1 unit/µl; Epicentre) (here named RNAP-σ70) were performed. Reactions were stopped with 50 µl of STOP buffer (2% SDS, 100 mM EDTA). Non-incorporated nucleotides were removed using MicroSpin^TM^ G-25 columns (GE Healthcare). Then, RNA was ethanol precipitated using Pellet Paint Co-Precipitant (Millipore), a visible dye-labelled carrier. The pellet was dissolved in 10 µl of loading buffer (80% formamide, 10 mM EDTA, pH 8.0, 0.1% xylene cyanol, 0.1% bromophenol blue), heated at 95°C for 5 min, and subjected to electrophoresis in 8 M urea-6% polyacrylamide gels. To estimate the size of the runoff transcripts, dideoxy-mediated chain termination sequencing reactions of a particular DNA were run in the same gel. Labelled products were visualized using a Fujifilm Image Analyzer FLA-3000. Single-stranded DNA molecules have been previously used to estimate chain lengths of RNA molecules by comparing their electrophoretic mobilities on polyacrylamide gels that contain a high concentration of urea (8 M) (del Solar et al., 1990; del Solar and Espinosa, 1992).

### Bioinformatics Analyses

The DNA sequence of gene *sigA* (*rpoD*) (1,110-nt) was extracted by selecting coordinates 965,281–966,390 from the *S. pneumoniae* R6 genome (NC_003098.1). A standard nucleotide Megablast (Altschul et al., 1990) was performed with the default parameters. Only representative strains of those *Streptococcus* species related to humans (pathogenic, commensal or probiotic) were selected from retrieved sequences. Sequences were then aligned in MEGA-X software (Kumar et al., 2018) using the ClustalW algorithm configured for the highest accuracy (with default settings). The phylogenetic tree was inferred by using the Maximum Likelihood method and the Tamura-Nei model (Tamura and Nei, 1993). The sequence of the SigA from *S. pneumoniae* R6 (identity entry: P0A4J0) was used for performing a search for homologous proteins in the Uniprot database (The UniProt Consortium, 2019). Proteins with similar lengths related to SigA from *S. pneumoniae* R6 were then aligned with Clustal Omega (Sievers and Higgins, 2014) as implemented in the UniprotKB tools, highlighting the sequence motifs and other regions of interest. The comparative analysis and three-dimensional modelling of the SigA were constructed with the SWISS-MODEL server (Waterhouse et al., 2018), by using the crystal structure of the *E. coli* holoenzyme as reference (https://swissmodel.expasy.org/templates/4lk1.1, chain F). The second-best scoring model, which used as the template *Bacillus subtilis* SigA structure from residue 100 to residue 371 (https://swissmodel.expasy.org/templates/7ckq.1, chain F), was also taken for the comparison to highlight structural conservation between SigA (RpoD) sigma factors core region (domains 2, 3, and 4). Additionally, a deep learning transform-restrained Rosetta (trRosetta) protein structure prediction server was used to validate the SWISS-MODEL results and compare the models obtained by both methods (Yang et al., 2020). trRosetta uses both homologous templates and *de novo* modelling through deep learning-based prediction to inter-residue orientations and distances coupled with Rosetta-based optimization method that combines the predicted restraints with components of the Rosetta energy minimization function. This approach let trRosetta outperform all previously described structure-prediction methods in the 13th Community-Wide Experiment on the Critical Assessment of Techniques for Protein Structure Prediction (CASP13)- and Continuous Automated Model Evaluation (CAMEO)-derived sets benchmark. The trRosetta-made model of SigA was built with restraints from both deep learning and homologous templates from *E. coli* (PDBs 6pst and 4yfk) and *Mycobacterium tuberculosis* (PDBs 6c05 and 5uh5).

## Results and Discussion

### The *sigA* Gene of *S. pneumoniae* R6

**FIGURE 1:**
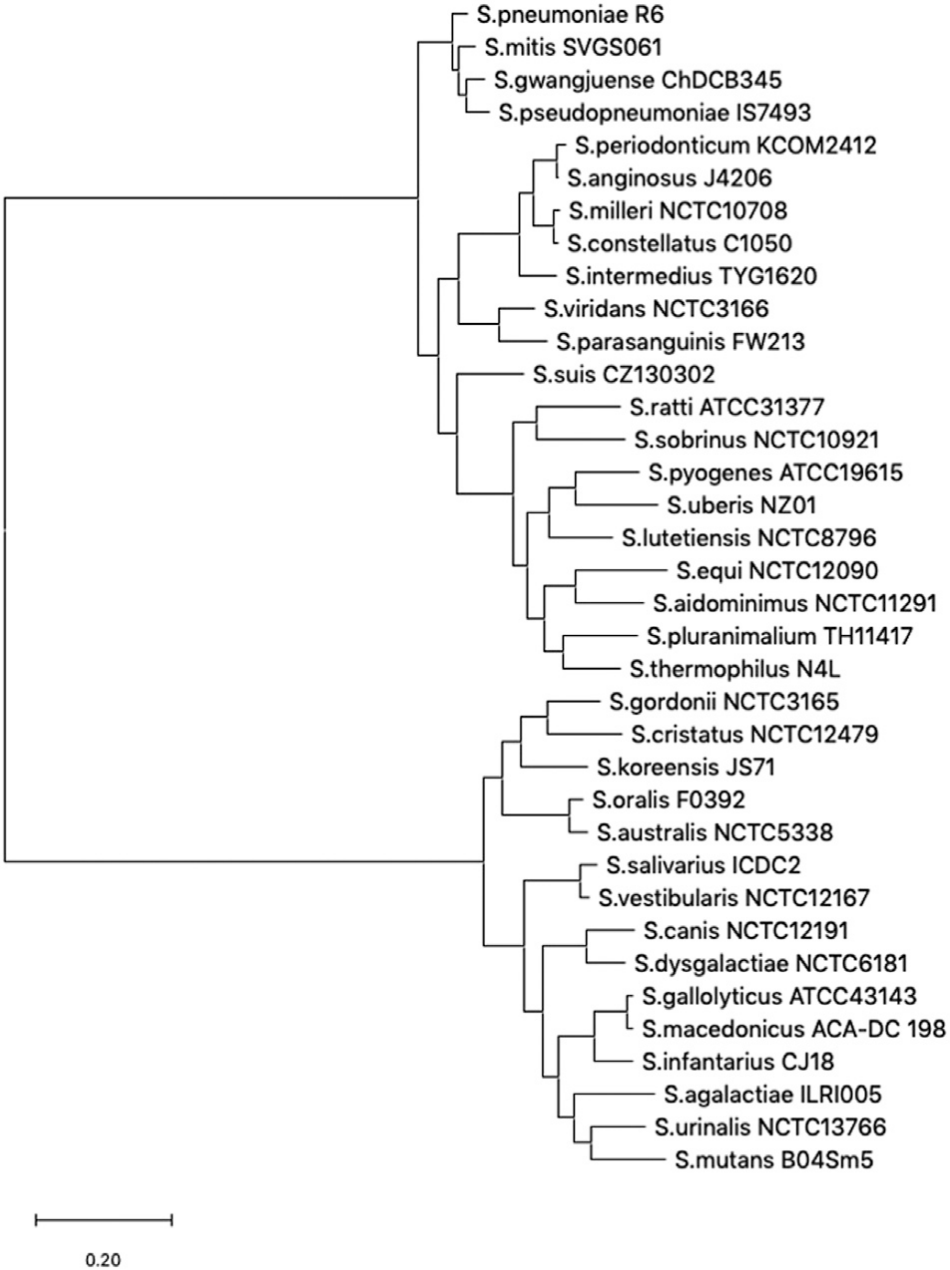
Phylogenetic tree of the *sigA* gene. Evolutionary analyses were conducted in MEGA X by comparing *sigA* gene sequences from the 36 representative *Streptococcus* species listed in Supplementary Table S1 (Supplementary Material). The tree with the highest log likelihood (−19,316.02) is shown. Initial tree(s) for the heuristic search were obtained automatically by applying Neighbor-Join and BioNJ algorithms to a matrix of pairwise distances estimated using the Tamura-Nei model, and then selecting the topology with a superior log-likelihood value. The tree is drawn to scale, with branch lengths measured in the number of substitutions per site.

In the genome of *S. pneumoniae* strain R6 (NC_003098.1) (Hoskins et al., 2001), the ATG codon at coordinate 965,281 is the translation start site of the *sigA* gene. Translation from this ATG codon generates a protein of 369 residues (P0A4J0). Using the *sigA* gene sequence of *S. pneumoniae* R6 as query (SPR_RS04905), we performed a Megablast analysis and selected 36 representative *Streptococcus* strains (Supplementary Table S1). The Phylogenetic tree of the *sigA* gene from such representative strains (Figure 1) showed two main clades. The *sigA* gene of *S. pneumoniae* was included in a subclade and found to be closely related to its homologs in the genomes of *S. mitis*, *S. gwangjuense* and *S. pseudopneumoniae* (mean base substitutions per site of 0.064).

**FIGURE 2:**
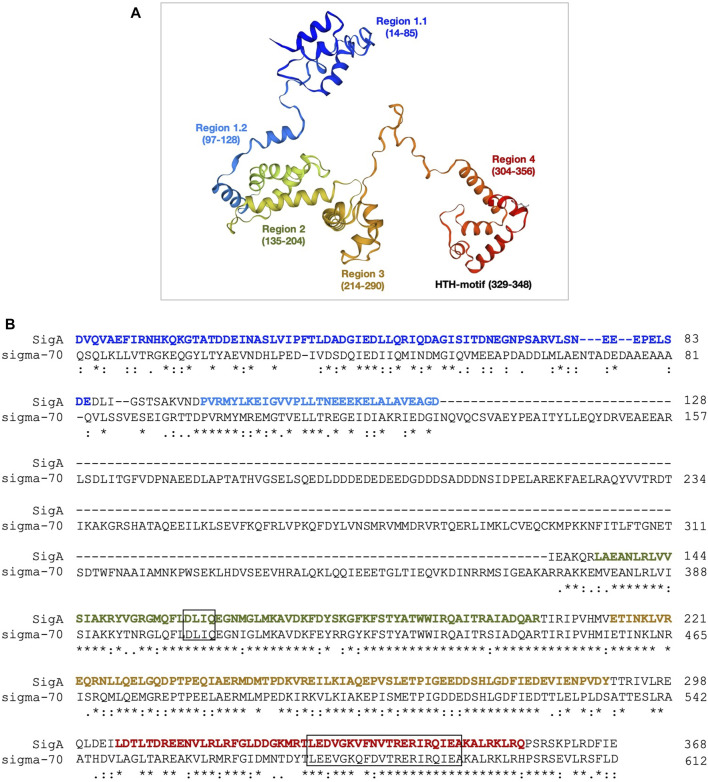
3D structure model of the SigA protein. **(A)** The homology model of SigA (P0A4J0) from *S. pneumoniae* strain R6 was constructed in the SWISS-MODEL server using the structure of the *E. coli* holoenzyme (RNAP-σ70) as the template. The model includes residue D12 to residue E368. Different regions are indicated. **(B)** Sequence alignment of the SigA and *E. coli* σ70 proteins. The DLIQ and HTH motifs are highlighted in boxes. The sequence colours correspond to the colours assigned to the regions shown in panel A. The region N128-A372 of *E. coli* σ70 is not present in SigA. The asterisks (*), colons (:) and dots (.) indicate identical amino acid residues, conserved substitutions, and semi-conserved substitutions, respectively.

We run BLAST to analyse the sequence conservation between the pneumococcal SigA factor and the housekeeping sigma factors σ70 from E*. coli* (613 residues) and SigA from *Bacillus subtilis* (371 residues). The analysis revealed that the essential core region (domains 2, 3, and 4) has a very high level of conservation between the pneumococcal SigA factor and the two other proteins, reaching 66% identity and 88% similarity for σ70 of *E. coli* and 80% identity and 92% similarity for SigA of *B. subtilis* (Supplementary Table S2 and Figure S1). We also used SWISS-MODEL and trRosetta (transform-restrained Rosetta) servers to obtain theoretical 3D models of the pneumococcal SigA factor (Waterhouse et al., 2018; Yang et al., 2020). The top model from SWISS-MODEL covered almost the entire sequence of the SigA protein, from residue D12 to residue E368 using the *E. coli* σ70 structure (PDB 4lk1) as the template (Figure 2A). As illustrated in Figure 2B, the main difference between both proteins is that the region from residue N128 to residue A372 of the σ70 factor is absent in SigA. Such a region is important for promoter escape and hinders early elongation pausing in σ70-like sigma factors (Shin et al., 2021). The second-best model from SWISS-MODEL used the *B. subtilis* SigA structure (PDB 7ckq), which due to flexibility of the domain 1.1 misses that region in the determined structure. Although 60% similarity (22% identity) for this N-terminal less conserved region (residues 1–96 of *S. pneumoniae* SigA) between the *E. coli* σ70 and the pneumococcal SigA sequences seems to provide sufficient similarity for homology modelling, we also used trRosetta server for complementary prediction, relying on its top scores at CASP13 experiment. The trRosetta model was obtained with restraints from both deep learning and homologous templates and supported the ones generated with SWISS-MODEL (superposition of models is shown in Supplementary Figure S2). The RMSD between both models equals 2.7 Å (Z score = 21.6) for the highly conserved core region (residues 97–369), and 4.0 Å (Z score = 4.3) for the N-terminal less conserved and mobile region, when comparing separated fragments at the DALI server (Holm, 2020). Submission of the complete models results in RMSD of 3.2 Å (Z score = 20.4) calculated over a region covering residues 80–369 of both models. Additionally, a superposition of SigA models on holoenzymes structures is shown in Supplementary Figure S3.

The 3D structure model of SigA let us visualize the protein functional regions assigned by sequence alignment at Pfam (Mistry et al., 2021) (Supplementary Figure S4, Figure 2): i) region 1.1 (residues 14–85) involved in the promoter selection; ii) region 2 (residues 135–204) that contains both the −10 promoter recognition helix and the primary core RNA polymerase binding determinant; iii) region 3 (residues 214–290) involved in binding to the core RNA polymerase; with a role in the recognition of some specific promoters containing an extended −10 element; and iv) region 4 (residues 304–356) involved in the binding to the −35 element. Out of the four regions, the most important domains are represented by region 2, in which the highly conserved DLIQ motif involved in the interaction with the β′ subunit (RpoC) is found; and region 4 containing a helix-turn-helix (HTH) motif that binds to the −35 element (Figure 2B). To study the degree of conservation of these motifs, 12 SigA proteins belonging to the σ70 factor SigA subfamily were selected from a total of 75 manually annotated (curated) protein sequences available in the Uniprot database. After performing a multiple sequence alignment, the results showed a high degree of diversity in region 1, whereas regions 2–4 were highly conserved among several Gram-positive bacteria, including *B. subtilis*, *Enterococcus faecalis*, *Staphylococcus aureus*, *S. mutans*, and *S. pneumoniae* (Supplementary Figure S5 and Table S3).

### Purification of the Pneumococcal SigA Protein

**FIGURE 3:**
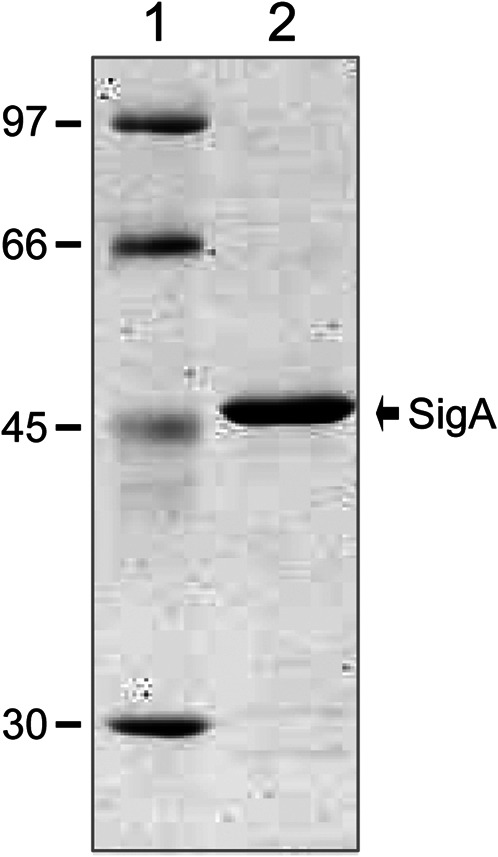
Purification of the pneumococcal SigA protein. Purified SigA was analysed by SDS-polyacrylamide (10%) gel electrophoresis. The gel was stained with Coomassie Blue. The molecular weight (in kDa) of proteins used as markers (GE Healthcare) is indicated on the left of the gel.

To overproduce the pneumococcal SigA protein (untagged version), we used an inducible expression system based on the pET24b plasmid, which carries the ϕ10-promoter recognized by the bacteriophage T7 RNA polymerase, and the *E. coli* BL21 (DE3) strain, which carries the T7 RNA polymerase-encoding gene fused to the *lacUV5* promoter (Studier and Moffatt, 1986) (see *Materials and Methods*). To purify SigA (369 amino acids), we set up a procedure that included the following steps: i) precipitation of nucleic acids with 0.2% PEI in the presence of 400 mM NaCl. SigA was recovered in the PEI pellet; ii) elution of SigA from the PEI pellet using a higher ionic strength buffer (700 mM NaCl); iii) after elution, proteins recovered in the supernatant (including SigA) were precipitated with 70% saturated ammonium sulphate; iv) chromatography on a heparin column equilibrated with a low ionic strength buffer (100 mM NaCl). About 80% of SigA was recovered when the column was washed with the same buffer, and v) chromatography on a gel filtration column. Both the equilibration buffer and the running buffer contained 100 mM NaCl. Purified SigA was analysed by SDS-polyacrylamide (10%) gel electrophoresis (Figure 3). The SigA preparation obtained after gel filtration chromatography was ∼95% pure. SigA (42.04 kDa, calculated from the predicted amino acid sequence) migrated slightly above the band corresponding to a 45 kDa standard protein.

### Recognition of the Pneumococcal *Pmga* Promoter by RNAP-SigA

**FIGURE 4:**
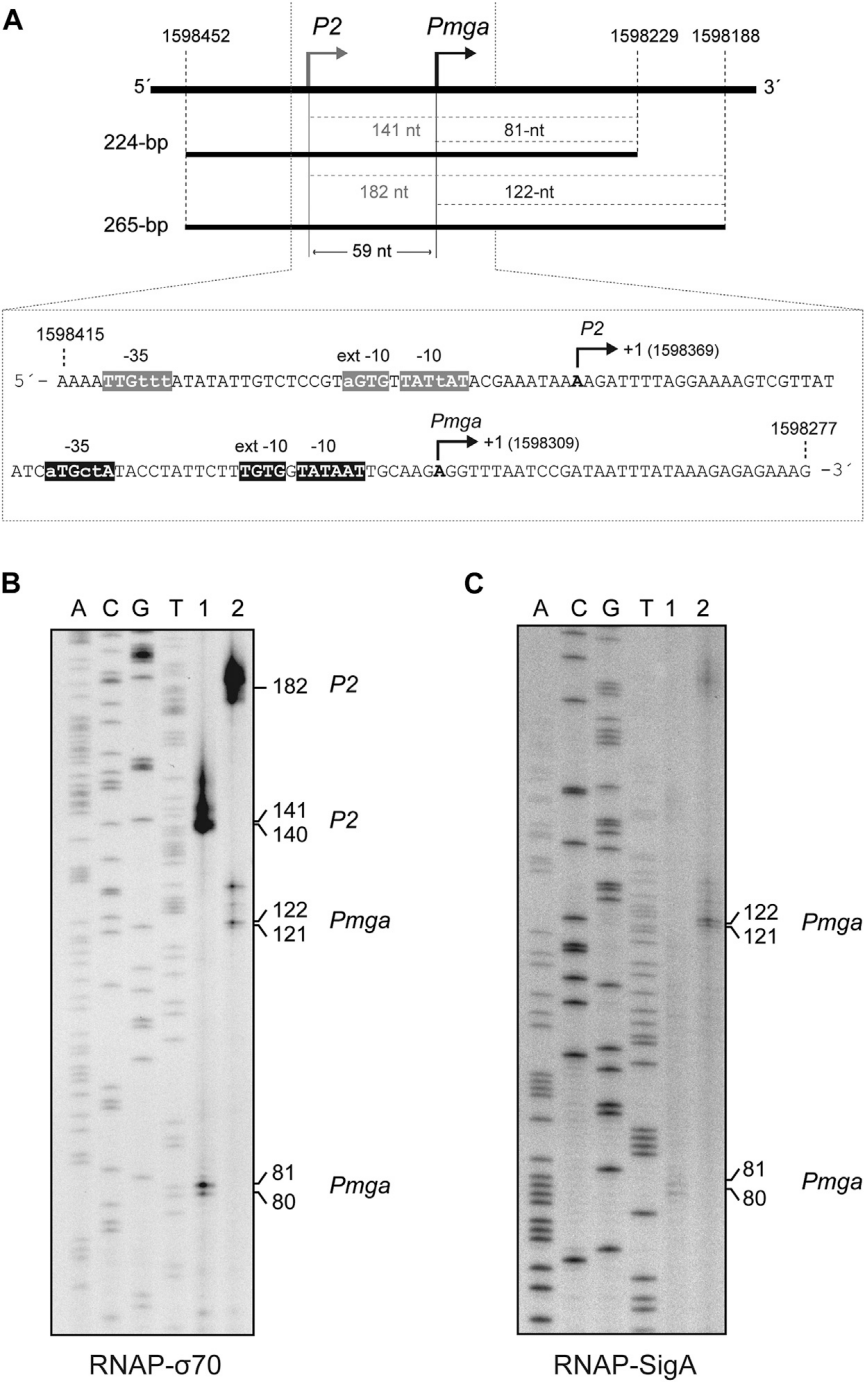
SigA recognizes the *Pmga* promoter. **(A)** The pneumococcal *Pmga* promoter. Upper part: Region of the pneumococcal R6 genome (Hoskins et al., 2001) that contains the *P2* promoter (this work) and the *Pmga* promoter (Solano-Collado et al., 2012). The coordinates of the 224-bp and 265-bp DNA fragments are indicated. Both DNA fragments were used for *in vitro* transcription assays (see Figures 4B,C). The size (in nucleotides) of the expected run-off transcripts is indicated. Lower part: Nucleotide sequence of the region spanning coordinates 1,598,415 and 1,598,277. The main sequence elements of the *P2* and *Pmga* promoters are indicated. Their transcription start site (position +1) is shown. **(B)** *In vitro* transcription experiments using RNAP-σ70. **(C)** *In vitro* transcription experiments using RNAP-SigA. In **(B)** and **(C)**, two DNA fragments of 224-bp (lanes 1) and 265-bp (lanes 2) were used as DNA templates (see Figure 4A). Denaturing gels (8 M urea-6% polyacrylamide) were used for resolving transcripts. Dideoxy-mediated chain termination sequencing reactions were run in the same gel (lanes A, C, G, T). In **(B)**, the sequencing reactions were prepared using a PCR-amplified fragment from the pneumococcal R6 genome (1,221-bp; coordinates 1,597,232–1,598,452) and the 5′-radiolabeled 1622D oligonucleotide (Table 1). Such a 1,221-bp DNA fragment was obtained by PCR using R6 chromosomal DNA as a template and the 1622A and 1622C oligonucleotides (Table 1). In **(C)**, the sequencing reactions were prepared using a PCR-amplified fragment from the R6 genome (421-bp; coordinates 1,598,229–1,598,649) and the 5′-radiolabeled 1622C oligonucleotide (Table 1). Such a 421-bp DNA fragment was obtained by PCR using R6 chromosomal DNA as a template and the 1622D and 1622B oligonucleotides (Table 1). The size (in nucleotides) of the transcription products is indicated on the right of the gels.

The pneumococcal *mgaSpn* gene encodes a transcriptional regulator (Mga*Spn*) (Solano-Collado et al., 2012; Solano-Collado et al., 2013). In the pneumococcal R6 genome (NCBI RefSeq NC_003098.1) (Hoskins et al., 2001), the ATG codon at coordinate 1,598,270 is likely the translation initiation codon of *mgaSpn*, as it is preceded by a putative Shine-Dalgarno sequence (5′-AAAGAGAGAAAG-3′) (coordinates 1,598,288–1,598,277) (Figure 4A). Previous *in vivo* experiments showed that i) the *mgaSpn* gene is transcribed in exponentially growing pneumococcal cells, ii) the transcription start site (position +1) of *mgaSpn* is located at coordinate 1,598,309, and iii) the promoter of *mgaSpn* (known as *Pmga*) has the features of a promoter recognized by a housekeeping sigma factor: a consensus −10 element (5′-TATAAT-3′), a consensus −10 extension element (5′-TGTG-3′), and shows a 3/6 match at the −35 element (5′-aTGctA-3′) (Solano-Collado et al., 2012) (see Figure 4A).

By electrophoretic mobility shift assays (Supplementary Figure S6), we determined that the holoenzyme constituted by the *E. coli* RNA polymerase core and the pneumococcal SigA factor (RNAP-SigA) was able to bind to linear DNA. We used a radioactively labelled 222-bp DNA fragment (coordinates 1,598,519–1,598,298 of the R6 genome), which contains the *P1623B* and *Pmga* divergent promoters (Solano-Collado et al., 2013). To further analyse whether RNAP-SigA was able to recognize specifically the *Pmga* promoter, we performed *in vitro* transcription experiments under multiple-round conditions using two linear DNA fragments of 224-bp (coordinates 1,598,452–1,598,229) and 265-bp (coordinates 1,598,452–1,598,188) as DNA templates (Figure 4A). Such fragments contain the *Pmga* promoter but not the *P1623B* promoter (Solano-Collado et al., 2013). Transcription from the *Pmga* promoter was expected to generate run-off transcripts of 81-nt (224-bp template) and 122-nt (265-bp template), respectively. The transcription products were resolved on denaturing gels (6% PAA, 8M urea) (Figure 4B,C), and their sizes were estimated taking into account that an RNA molecule migrates slightly slower than a DNA molecule of the same size (Sambrook et al., 1989). Using RNAP-SigA, transcripts of the expected size were detected with both the 224-bp template and the 265-bp template (Figure 4C, lanes 1 and 2). However, in addition to such products, transcripts of 141-nt (224-bp template) and 182-nt (265-bp template) were detected using the *E. coli* RNA polymerase holoenzyme that contains the housekeeping σ70 factor (RNAP-σ70) (Figure 4B, lanes 1 and 2). This result indicated that RNAP-σ70 was able to recognize, in addition to the *Pmga* promoter, a sequence (here named *P2*) located upstream of *Pmga* and to initiate transcription at coordinate 1,598,369 (see Figure 4A). The *P2* promoter sequence has a near-consensus −10 element (5′-TATtAT-3′), a near-consensus −10 extension element (5′-a**GTG**-3′), and a 3/6 match at the −35 element (5′-TTGttt-3′). The −10 and −35 regions are separated by 20-nt. Therefore, we conclude that the pneumococcal SigA factor recognizes mainly the *Pmga* promoter. Upstream of this promoter, there are sequence motifs that can be recognized by RNAP-σ70.

### Recognition of the *Pcr* Promoter of pMV158 by RNAP-SigA

**FIGURE 5:**
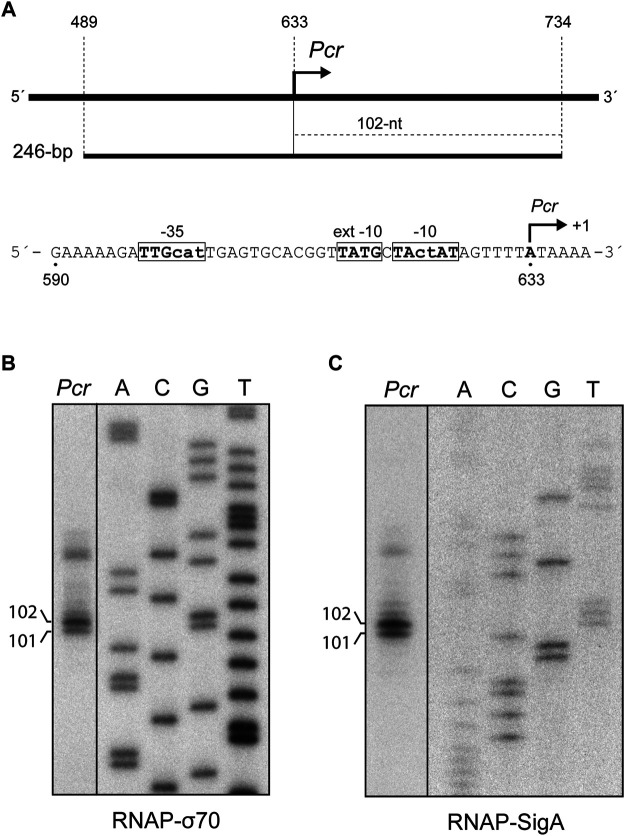
SigA recognizes the *Pcr* promoter. **(A)** Region of the pMV158 plasmid that contains the *Pcr* promoter (del Solar et al., 1990; del Solar and Espinosa, 1992). The coordinates of the 246-bp DNA fragment are indicated. This fragment was used for *in vitro* transcription assays (see Figures 5B,C). The size of the expected run-off transcript is indicated. The main sequence elements of the *Pcr* promoter, including the transcription start site (position +1), are shown. **(B)** *In vitro* transcription assays using RNAP-σ70. **(C)** *In vitro* transcription assays using RNAP-SigA. In **(B)** and **(C)**, the 246-bp DNA fragment was used as the DNA template. Denaturing gels (8 M urea-6% polyacrylamide) were used for resolving transcripts. Dideoxy-mediated chain termination sequencing reactions were run in the same gel (lanes A, C, G, T). All the lanes displayed came from the same gel (delineation with dividing lines). Sequencing reactions were prepared **(B)** using M13mp18 DNA and the 5′-radiolabeled-40 M13 oligonucleotide (Table 1; Yanisch-Perron et al., 1985) or **(C)** using a *Sau*3AI DNA fragment from plasmid pKN1562 and the 5′-radiolabeled oligo-2 oligonucleotide (Table 1; Monti et al., 2007). The size (in nucleotides) of the transcription products is indicated on the left of the gels.

Plasmid pMV158 (5,540 bp) replicates by the rolling circle mechanism and was isolated from *S. agalactiae*. However, it replicates in many bacterial species, including *S. pneumoniae* and *E. coli*, although at different copy numbers, around 30 in the pneumococcus and seven in the Gram-negative host (Lacks et al., 1986; del Solar et al., 1997; Lorenzo-Díaz and Espinosa, 2009; Lorenzo-Díaz et al., 2017). The *Pcr* promoter directs the synthesis of a bicistronic mRNA, which encodes a transcriptional repressor involved in plasmid copy-number control, CopG, and a replication initiator protein, RepB. Promoter *Pcr* has been characterized by *in vivo* and *in vitro* assays. Specifically, using total RNA from *S. pneumoniae* cells harbouring a pMV158-derivative, the *copG-repB* transcription start site was identified at coordinate 633 of pMV158 (del Solar et al., 1990). This conclusion was further confirmed by *in vitro* transcription experiments using the *E. coli* RNA polymerase holoenzyme that contains σ70 (del Solar et al., 1990; del Solar and Espinosa, 1992). Compared to the consensus sequence of the promoters recognized by the *E. coli* σ70 factor, the *Pcr* promoter has a 4/6 match at the −10 element (5′-TActAT-3′), a consensus −10 extension element (5′-TATG-3′), and a 3/6 match at the −35 element (5′-TTGcat-3′) (see Figure 5A).

To know whether the reconstituted RNAP-SigA enzyme was able to initiate transcription from the *Pcr* promoter, we performed *in vitro* transcription assays. A 246-bp DNA fragment (coordinates 489–734 of plasmid pMV158) was used as the DNA template (Figure 5A). As shown in Figure 5C, two main RNAs of about 101-nt and 102-nt were synthesized. Both products were also detected in assays performed with RNAP-σ70 (Figure 5B). The synthesized RNAs have the size expected for run-off transcripts initiated at coordinates 633 and 634, respectively (see Figure 5A). Thus, we conclude that the pneumococcal SigA factor can recognize the *Pcr* promoter of pMV158.

### Recognition of the *Pmob1* and *Pmob2* Promoters of pMV158 by RNAP-SigA

**FIGURE 6:**
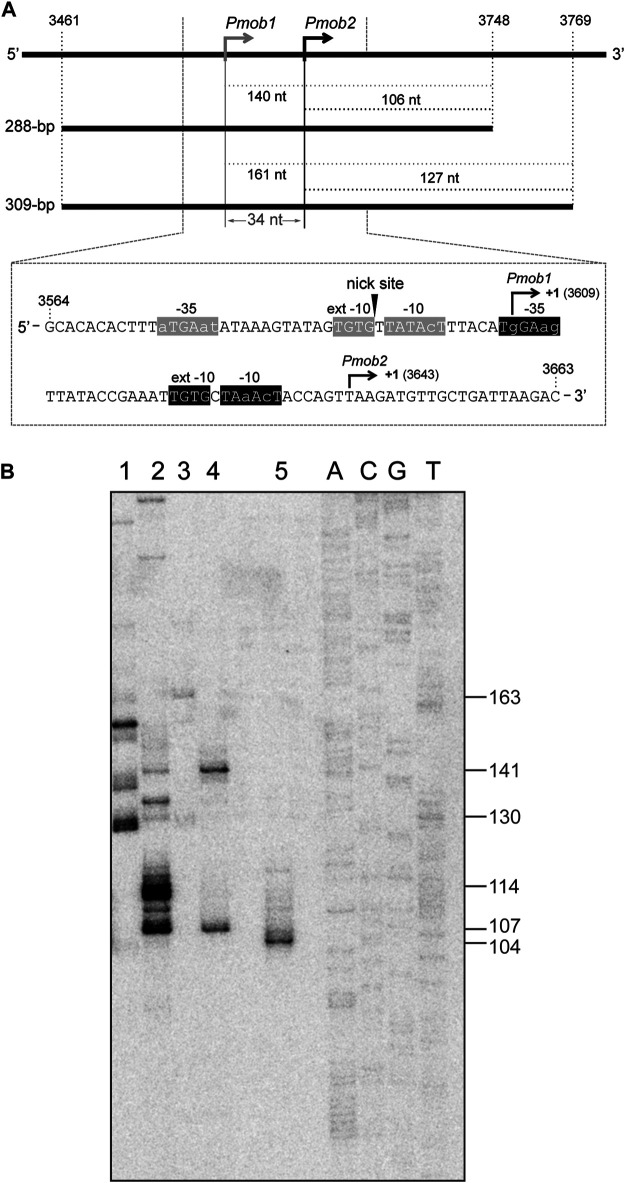
SigA recognizes the *Pmob1* and *Pmob2* promoters. **(A)** Region of the pMV158 plasmid that contains the *Pmob1* and *Pmob2* promoters (Farías et al., 1999; Lorenzo-Díaz et al., 2012). Upper part: the coordinates of the 288-bp and 309-bp DNA fragments are indicated. Both DNA fragments were used for *in vitro* transcription assays (see Figure 6B). The size (in nucleotides) of the expected run-off transcripts is indicated. Lower part: the nucleotide sequence of the region spanning coordinates 3,564 and 3,663 of pMV158 is shown. The arrowhead indicates the nick site. The main sequence elements of the *Pmob1* and *Pmob2* promoters are indicated. Their transcription start site (position +1) is shown. **(B)** *In vitro* transcription assays using RNAP-σ70 and RNAP-SigA. The 288-bp and 309-bp DNA fragments shown in Figure 6A were used as DNA templates. A denaturing gel (8 M urea-6% polyacrylamide) was used for resolving transcripts. Lane 1: 309-bp DNA template and RNAP-σ70. Lane 2: 288-bp DNA template and RNAP-σ70. Lane 3: 309-bp DNA template and RNAP-SigA. Lane 4: 288-bp DNA template and RNAP-SigA. Lane 5: transcript of 102-nt that comigrates with the 104-nt DNA marker. Dideoxy-mediated chain termination sequencing reactions were run in the same gel (lanes A, C, G, T). Sequencing reactions were prepared using a PCR-amplified fragment from the pneumococcal ST556 genome (1,009-bp IR1.2 fragment; oligonucleotides pr14686/pr15159) and the 5′-radiolabeled pr14687 oligonucleotide (Table 1) (see Li et al., 2020). The size (in nucleotides) of the DNA molecules that comigrate with the runoff transcripts is indicated on the right of the gel.

Although the streptococcal broad host range plasmid pMV158 is not conjugative, it can be mobilized among a wide number of bacterial species by the activity of the plasmid-encoded MobM protein, which is the prototype of the MOB_V1_ family of relaxases (Garcillán-Barcia et al., 2009; Fernández-López et al., 2014; Lorenzo-Díaz et al., 2014). Plasmids of the *Inc18* family, such as pIP501 or pAMβ1, are considered auxiliary plasmids that provide the machinery (coupling protein and the T4 secretion system; reviewed by Grohmann et al., 2018) for the conjugative mobilization of pMV158 (Priebe and Lacks, 1989). The mobilization module of pMV158 includes the *mobM* gene and the origin of transfer, *oriT*. To initiate the conjugative mobilization, MobM binds to *oriT* on supercoiled plasmid DNA and performs a histidine-mediated nucleophilic attack at the phosphate group of the specific dinucleotide 5′-GpT-3´ (coordinates 3,595–3,596; nick site) (Guzmán and Espinosa, 1997; Pluta et al., 2017). By *in vitro* studies, the minimal *oriT* sequence was delimited to a stretch of 26 nucleotides (coordinates 3,570–3,595) that is located just upstream of the nick site (Lorenzo-Díaz et al., 2011) (see Figure 6A). In addition to its role as initiator of transfer, MobM self-regulates its synthesis at the transcriptional level (Lorenzo-Díaz et al., 2012) and participates in the regulation of the plasmid copy number (Lorenzo-Díaz et al., 2017), contributing to the promiscuity of pMV158.

Studies on the transcription of the *mobM* gene led to the identification of two promoters, *Pmob1* and *Pmob2* (Figure 6A). Specifically, in *Lactococcus lactis*, the transcription start site of the *mobM* gene was located at coordinate 3,609 (promoter *Pmob1*) (Farías et al., 1999), whereas the *E. coli* RNA polymerase was shown to initiate transcription of *mobM* preferentially at coordinate 3,643 (promoter *Pmob2*) (Lorenzo-Díaz et al., 2012). Both promoters are subjected to self-regulation by MobM protein (Lorenzo-Díaz et al., 2012). As shown in Figure 6A, the *Pmob1* promoter has a near-consensus −10 element (5′-TATAcT-3′), a consensus −10 extension element (5′-TGTG-3′) and shows a 3/6 match at the −35 element (5′-aTGAat-3′). Additionally, this promoter is located within the *oriT* sequence, being its −10 element adjacent to the nick site. The *Pmob2* promoter, which is placed just downstream of the *Pmob1* promoter, shows a 4/6 match at the −10 element (5′-TAaAcT-3′), has a consensus −10 extension (5′-TGTG-3′) and shows a 3/6 match at the −35 element (5′-TgGAag-3′). The organization of the *mobM* promoter region has been considered a strategy of the promiscuous plasmid pMV158 to cope with the different types of transcription machinery of Gram-positive and Gram-negative bacteria (Lorenzo-Díaz et al., 2012).

In this study, we investigated whether the pneumococcal SigA factor was able to recognize the *Pmob1* and *Pmob2* promoters. Specifically, we performed *in vitro* transcription assays under multiple-round conditions using RNAP-SigA and two linear DNA fragments of 288-bp (coordinates 3,461–3,748) and 309-bp (coordinates 3,461–3,769) (Figure 6A). Depending on the promoter recognized by RNAP-SigA, runoff transcripts of 140-nt (*Pmob1*) or 106-nt (*Pmob2*) should be synthesized using the 288-bp DNA template, whereas runoff transcripts of 161-nt (*Pmob1*) or 127-nt (*Pmob2*) should be synthesized using the 309-bp DNA template. The *in vitro* transcription products were resolved on a denaturing gel (Figure 6B). As an internal control, a transcript of 102-nt was run in the same gel (Figure 6B, lane 5). This transcript comigrated with the 104-nt DNA marker, confirming that an RNA molecule migrates slightly more slowly than a DNA molecule of the same length on a denaturing polyacrylamide gel (Sambrook et al., 1989). Regarding RNAP-SigA, with the 288-bp DNA template (lane 4), two RNA products that comigrated with the 141-nt and 107-nt DNA markers were detected, indicating that RNAP-SigA was able to initiate transcription from both promoters, *Pmob1* and *Pmob2*. This conclusion was confirmed with the 309-bp DNA template (lane 3). In this case, two RNA products that comigrated with the 163-nt and 130-nt DNA markers were detected. Concerning RNAP-σ70, the RNA product that corresponds to transcription initiated at the *Pmob2* promoter was detected using the 288-bp DNA template (lane 2), as well as the 309-bp DNA template (lane 1). Moreover, RNA products that do not correspond to *Pmob1*-initiated events were detected. Taken together, we conclude that the pneumococcal SigA factor can recognize the *Pmob1* and *Pmob2* promoters, which ensures the conjugative mobilization of pMV158 from *S. pneumoniae* to other bacterial species.

## Conclusion

Definitive identification of promoters involves the use of various experimental approaches, including *in vitro* transcription (Ross and Gourse, 2009). In bacteria, four different subunits (β, β′, two copies of α and ω) form the structurally conserved RNA polymerase core, which transiently binds to a member of the sigma factor family forming the holoenzyme. As pointed out (Griesenbeck et al., 2017), even though there is only a low sequence identity among the core subunits across the domains of life, there is a high degree of structural conservation. To date, the pneumococcal sigma factor SigA has been poorly studied. Recently, it has been reported that a His-tagged SigA factor bound to the *E. coli* RNA polymerase core was able to recognize the promoter region of the pneumococcal *amiA* gene, a housekeeping gene required for log-phase growth (Inniss and Morrison, 2020). Here we have set up for the first time a procedure to purify an untagged version of SigA and analysed its functionality using the *E. coli* core enzyme and particular streptococcal promoters. We have shown that SigA recognizes *in vitro* the pneumococcal *Pmga* promoter, which had been defined previously using promoter-reporter fusions and primer extension assays (Solano-Collado et al., 2012). Furthermore, we have shown that SigA provides the RNA polymerase with the ability to transcribe genes involved in replication (*copG-repB*) and conjugative mobilization (*mobM*) of the *S. agalactiae* plasmid pMV158. Thus, SigA contributes to the broad-host-range of this plasmid, and consequently to the spread of antibiotic-resistance genes among bacteria. Finally, our bioinformatics analyses have revealed that essential SigA domains (binding to the core enzyme and binding to DNA) are highly conserved among relevant Gram-positive bacteria, including staphylococcal and enterococcal pathogens.

## Data Availability

The original contributions presented in the study are included in the article/Supplementary Material, further inquiries can be directed to the corresponding authors.

## Abbreviations

HTH, helix-turn-helix; IPTG, isopropyl β-D-1-thiogalactopyranoside; Km, kanamycin; nt, nucleotides; OD600, optical density at 600 nm; PAA, polyacrylamide; PEI, polyethyleneimine; RNAP-σ70, *E. coli* RNA polymerase holoenzyme; RNAP-SigA, *E. coli* RNA polymerase core and pneumococcal SigA factor; TY, tryptone-yeast extract.
