# Supplementary material for: Recognition of Streptococcal Promoters by the Pneumococcal SigA Protein
Source: Front Mol Biosci. 2021 Jun 24;8:666504. doi: 10.3389/fmolb.2021.666504 (PMC8264293; doi:10.3389/fmolb.2021.666504)
Supplement: Supplementary file 1 [file DataSheet1.PDF]

SUPPLEMENTARY MATERIAL

Recognition of Streptococcal Promoters by the Pneumococcal SigA Protein

Virtu Solano-Collado[1], Sofía Ruiz-Cruz[2], Fabián Lorenzo-Díaz[3], Radoslaw Pluta[4], Manuel Espinosa[5]* and Alicia Bravo[5]*

[1] Institute of Medical Sciences, University of Aberdeen, Aberdeen, United Kingdom.

[2] School of Microbiology, University College Cork, Cork, Ireland.

[3] Departamento de Bioquímica, Microbiología, Biología Celular y Genética, Universidad de La Laguna, Tenerife, Spain.

[4] Institute for Research in Biomedicine (IRB Barcelona), The Barcelona Institute of Science and Technology, Barcelona, Spain.

[5] Centro de Investigaciones Biológicas Margarita Salas, Consejo Superior de Investigaciones Científicas, Madrid, Spain.

* Corresponding authors

mespinosa@cib.csic.es (Manuel Espinosa); abravo@cib.csic.es (Alicia Bravo)

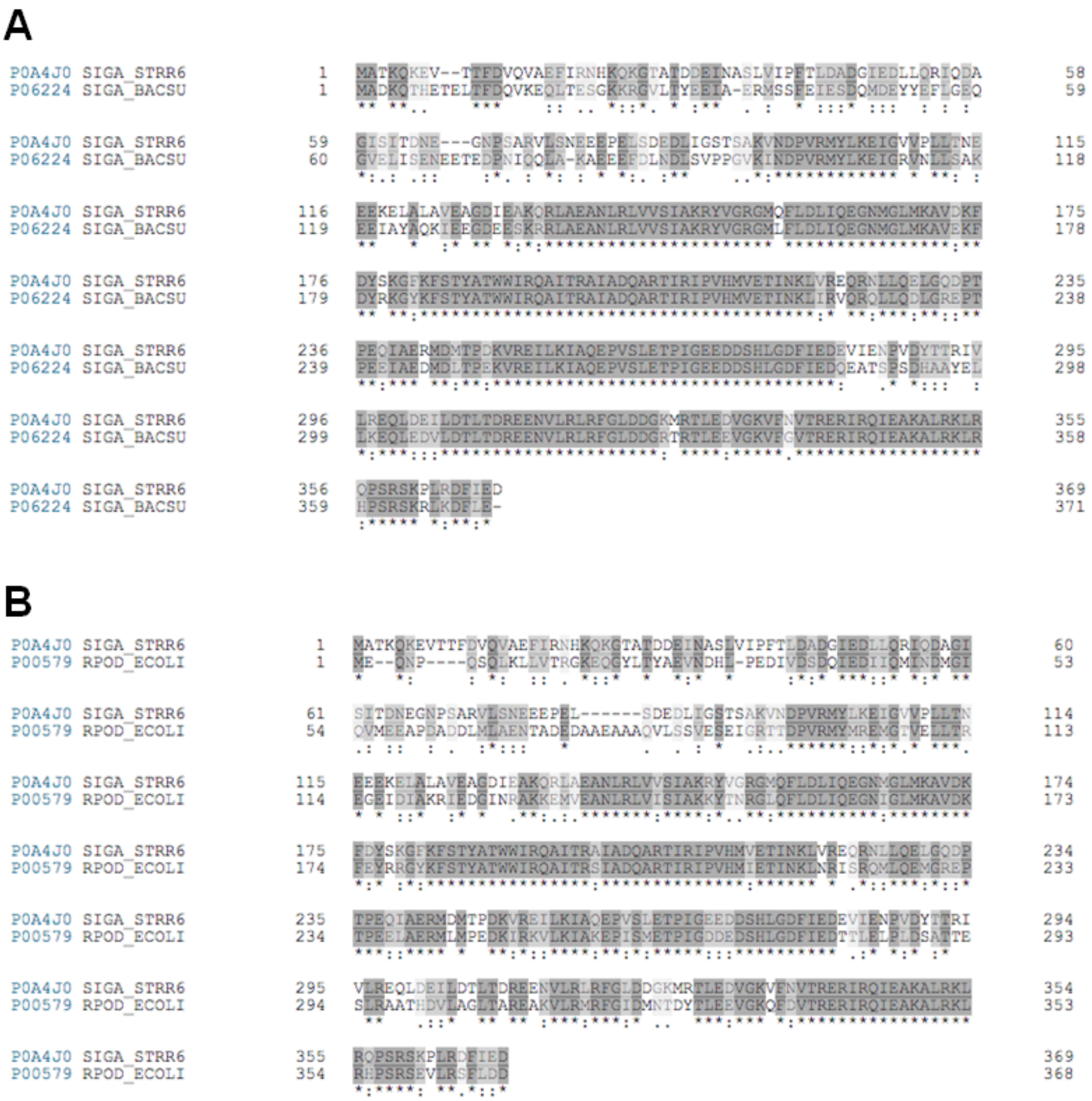

Figure S1. Protein sequence alignments. (A) Alignment of *Sp*-SigA (pneumococcal SigA factor) and *Bs*-SigA (SigA from *B. subtilis*). **(B)** Alignment of *Sp*-SigA and *Ec*-σ70 (σ70 from *E. coli* lacking the non-conserved region σNCR_128-372, from residue N128 to residue A372) (Shin et al., 2021). The asterisks (*), colons (:) and dots (.) indicate identical amino acid residues, conserved substitutions, and semi-conserved substitutions, respectively.

Reference

Shin, Y., Qayyum, M. Z., Pupov, D., Esyunina, D., Kulbachinskiy, A., and Murakami, K. S. (2021). Structural basis of ribosomal RNA transcription regulation. *Nat. Commun.* 12, 528. doi: 101038/s41467-020-20776-y

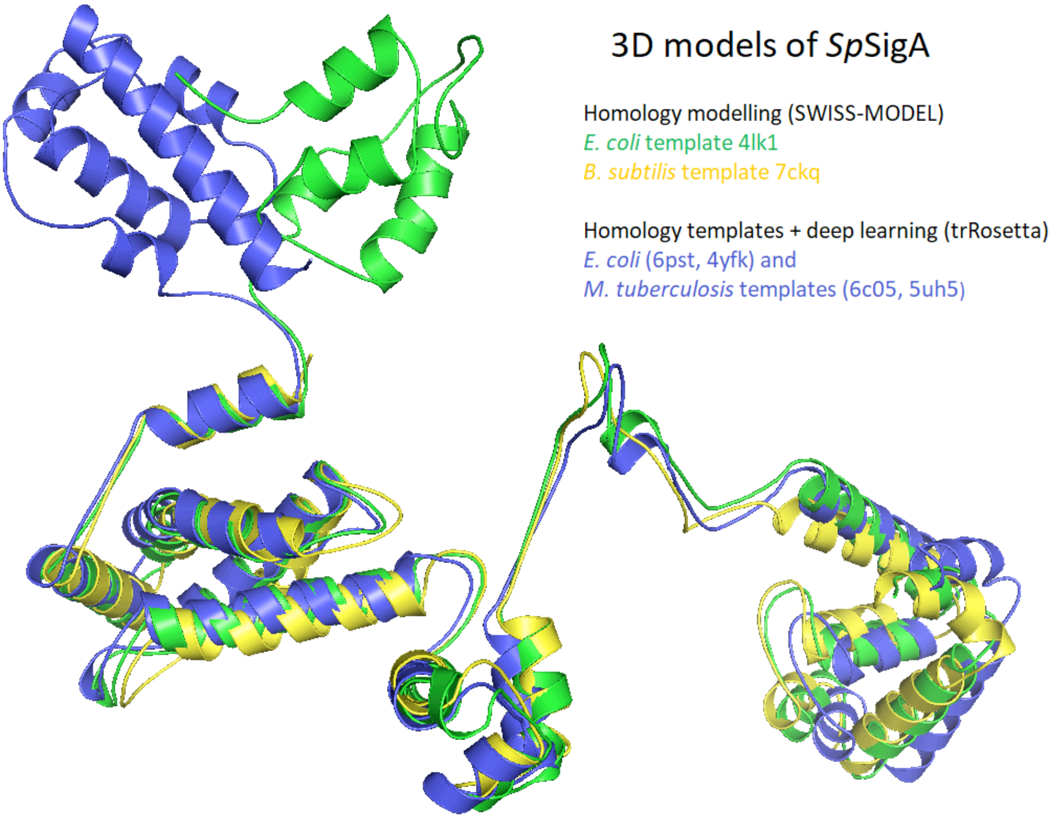

Figure S2. Superposition of SigA 3D models. The models were generated by the SWISS-MODEL homology modelling server and by the trRosetta deep learning and homology modelling server. The superposition reflects the mobility of the N-terminal region that precedes domain 2 and highlights conformational conservation of the core region (domains 2, 3, and 4) of RNA polymerase-bound sigma factors.

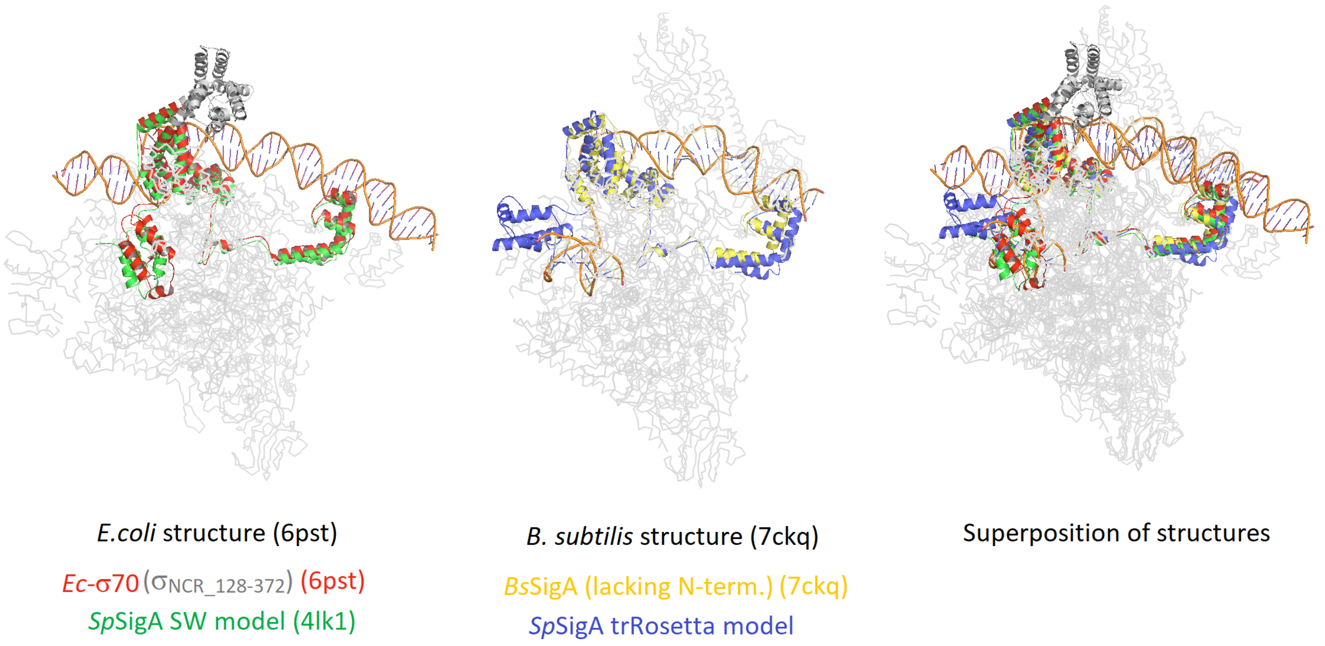

Figure S3. Superposition of SigA models on holoenzymes structures. Sigma factors and DNA are shown in cartoon representation, and other proteins are shown as light grey ribbons. The top SWISS-MODEL (SW) model of the pneumococcal SigA factor is shown in green and the top model from trRosetta is shown in blue. The N-terminal region of the sigma factors needs to relocate to allow DNA binding in the transcription-competent complex.

A

RNA polymerase sigma factor SigA {ECO:0000255|HAMAP-Rule:MF_00963}
Streptococcus pneumoniae (strain ATCC BAA-255 / R6) (NCBI taxonomy ID 171101)
369 amino acids

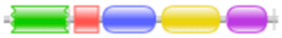

Source	Domain	Start	End
Pfam	Sigma70_r1_1	10	87
disorder	n/a	24	26
disorder	n/a	66	89
Pfam	Sigma70_r1_2	97	128
low_complexity	n/a	115	126
Pfam	Sigma70_r2	135	205
Pfam	Sigma70_r3	214	291
disorder	n/a	230	244
disorder	n/a	265	267
Pfam	Sigma70_r4	303	356
disorder	n/a	365	366

B

RNA polymerase sigma factor SigA {ECO:0000255|HAMAP-Rule:MF_00963}
Bacillus subtilis (strain 168) (NCBI taxonomy ID 224308)
371 amino acids

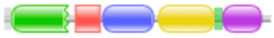

Source	Domain	Start	End
disorder	n/a	1	18
Pfam	Sigma70_r1_1	10	89
disorder	n/a	40	41
disorder	n/a	63	80
Pfam	Sigma70_r1_2	100	133
Pfam	Sigma70_r2	138	208
Pfam	Sigma70_r3	217	294
disorder	n/a	234	236
disorder	n/a	238	247
disorder	n/a	268	270
disorder	n/a	275	286
disorder	n/a	292	295
coiled_coil	n/a	293	320
Pfam	Sigma70_r4	306	359

C

RNA polymerase sigma factor RpoD {ECO:0000255|HAMAP-Rule:MF_00963}
Escherichia coli (strain K12) (NCBI taxonomy ID 83333)
613 amino acids

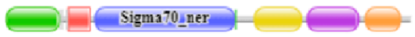

Source	Domain	Start	End
Pfam	Sigma70_r1_1	3	83
disorder	n/a	3	10
disorder	n/a	23	24
disorder	n/a	28	29
disorder	n/a	32	33
disorder	n/a	35	36
disorder	n/a	59	73
low_complexity	n/a	72	81
disorder	n/a	77	88
disorder	n/a	94	99
Pfam	Sigma70_r1_2	96	128
disorder	n/a	103	105
Pfam	Sigma70_ner	137	348
disorder	n/a	159	216
low_complexity	n/a	188	209
coiled_coil	n/a	330	350
Pfam	Sigma70_r2	379	449
Pfam	Sigma70_r3	458	535
disorder	n/a	468	486
disorder	n/a	509	511
disorder	n/a	513	514
disorder	n/a	534	535
Pfam	Sigma70_r4	547	600
coiled_coil	n/a	573	593

Figure S4. Protein architecture of housekeeping sigma factors from (A) *S. pneumoniae*, **(B)** *B. subtilis*, and **(C)** *E. coli*. Snapshots from the Pfam database (Mistry et al., 2021).

Reference

Mistry, J., Chuguransky, S., Williams, L., Qureshi, M., Salazar, G. A., Sonnhammer, E. L. L., Tosatto, S. C. E., Paladin, L., Raj, S., Richardson, L. J., Finn, R. D., and Bateman, A. (2021). Pfam: The protein families database in 2021. *Nucleic Acids Res.* 49, D412-D419. doi: 10.1093/nar/gkaa913

```
sp|P33656|SIGA_CLOAB      MKNKTEVKNGGEKKNSKKVSKEESAKEKNEKMKIVKNLIDKGKKSGSLTYKEIMDEL-QE   59
sp|Q04506|SIGA_LACLA      MDSPK-------TLARVEVKENGDTFNVKAYEKAVKSYITKRKPLGEALDEEIMDELSVK   53
sp|P58290|SIGA_LACLC      -----------------------------AYEKAVKGYITKRKPLGEALDEEIMDELSVK   31
sp|O33662|SIGA_STRMU      -------------------MVNNKKKTSSTFNVQVADFIRNHKKEGVAVDDEVTEKLVIP   41
sp|P0A4J0|SIGA_STRR6      --------------------MATKQKEVTTFDVQVAEFIRNHKQKGTATDDEINASLVIP   40
sp|P52329|SIGA_ENTFA      ----------------------MEKETSKKYEAAVAAFIKENKPKGTVVYDDLANQLATP   38
sp|Q8CP24|SIGA_STAES      MSDNQ-------VK-----IKKQTIDPTLTLEDVKKQLIDKGKKEGHLSHEEIAEKL-QN   47
sp|Q99TT5|SIGA_STAAN      MSDNT-------VK-----IKKQTIDPTLTLEDVKKQLIEKGKKEGHLSHEEIAEKL-QN   47
sp|Q92BQ6|SIGA_LISIN      MSD----------K-----TQNTKPVAELNVEQVKEALIEEGKKKGILTYAKIAARL-AP   44
sp|P52331|SIGA_LISMO      MSD----------K-----TKNTKPVAELSVEQVKEALIEEGKKKGILTYAKIAARL-AP   44
sp|O66381|SIGA_BACHD      MAE----------K-----PLRPLAEGELSIDQVKEQLVELGKKRGVLTYAEITEKL-AP   44
sp|P06224|SIGA_BACSU      -------------M-----ADKQTHETELTFDQVKEQLTESGKKRGVLTYEEIAERM-SS   41
                                                                    *  *     .:   :

sp|P33656|SIGA_CLOAB      VDLGPEQIEKIYEVLESVGVDVVGDMHEIE--------VEEE---DLDL-TIPEGIAIDD   107
sp|Q04506|SIGA_LACLA      FGIVDDALEDLFKQIQDAGISIVDKDGNPSPLALVTDEIEKEEVSDTAMDEIVTNVRIDD   113
sp|P58290|SIGA_LACLC      FGIVDDALEDLFKQIQDAGISIVDKDGNPSPLALVTDEIEKEEVSDTAMDEIVTNVRIDD   91
sp|O33662|SIGA_STRMU      FELEAEQIDDLLERLTDGGISITDREGNPSTKYAVE-EIKPEELTDEEL-LGSNSAKVND   99
sp|P0A4J0|SIGA_STRR6      FTLDADGIEDLLQRIQDAGISITDNEGNPSARVLSN-EEEP-ELSDEDL-IGSTSAKVND   97
sp|P52329|SIGA_ENTFA      YTLDAEAMEKLIQKVEDAGISVVDENGDPSEHSLKKDEKEAEKAQAEDL-SAPTGVKIND   97
sp|Q8CP24|SIGA_STAES      FEMDSDQMDDFFDQLNDNDITLVNEKDSSD---------TDDKINPNDL-SAPPGVKIND   97
sp|Q99TT5|SIGA_STAAN      FDIDSDQMDDFFDQLNDNDISLVNEKDSSD---------TDEKLNPSDL-SAPPGVKIND   97
sp|Q92BQ6|SIGA_LISIN      FTLDSDQMDEYLEHVGEAGIEVSDDADDEDPDETELVKEETESFDLTDM-SVPPGVKIND   103
sp|P52331|SIGA_LISMO      FTLDSDQMDEYLEHVGEAGIEVSDDADDEDPDETELVKEETESFDLTDM-SVPPGVKIND   103
sp|O66381|SIGA_BACHD      YDQDSDQMDEFFEYLGEQGVEILNDNEE--VPSLQQVEKEEEEFDLNDL-SVPPGVKIND   101
sp|P06224|SIGA_BACSU      FEIESDQMDEYYEFLGEQGVELISENEETEDPNIQQLAKAEEEFDLNDL-SVPPGVKIND   100
                               : ::.  . : . .: : .   .                    :     .  ::*

sp|P33656|SIGA_CLOAB      PVRMYLKEIGKVPLLSPEEEIDLAQRIKNG---DRSARKKLAEANLRLVVSIAKRYVGRG   164
sp|Q04506|SIGA_LACLA      PVRMYLKEIGRYPLISLDEETKLAEAIIAGGEGAEFAKQMLAEANLRLVVSIAKRYSGRG   173
sp|P58290|SIGA_LACLC      PVRMYLKEIGRYPLISLDEETKLAEAIIAGGEGAEFAKQMLAEANLRLVVSIAKRYSGRG   151
sp|O33662|SIGA_STRMU      PVRMYLKEIGVVPLLTNEEEKELAIAVENG---DLEAKQRLAEANLRLVVSIAKRYVGRG   156
sp|P0A4J0|SIGA_STRR6      PVRMYLKEIGVVPLLTNEEEKELALAVEAG---DIEAKQRLAEANLRLVVSIAKRYVGRG   154
sp|P52329|SIGA_ENTFA      PVRMYLKEIGRVQLLTAAEEVELALKIEEG---DQEAKQRLAEANLRLVVSIAKRYVGRG   154
sp|Q8CP24|SIGA_STAES      PVRMYLKEIGRVNLLSAQEEIELAKRIEQG---DEIAKSRLAEANLRLVVSIAKRYVGRG   154
sp|Q99TT5|SIGA_STAAN      PVRMYLKEIGRVNLLSAQEEIELAKRIEQG---DEVAKSRLAEANLRLVVSIAKRYVGRG   154
sp|Q92BQ6|SIGA_LISIN      PVRMYLKEIGRVDLLTADEEIALAKRIEAG---DIEAKGRLAEANLRLVVSIAKRYVGRG   160
sp|P52331|SIGA_LISMO      PVRMYLKEIGRVDLLTADEEIALAKRIEAG---DIEAKGRLAEANLRLVVSIAKRYVGRG   160
sp|O66381|SIGA_BACHD      PVRMYLKEIGRVPLLTAEEEIELATRIEQG---DEEAKRRLAEANLRLVVSIAKRYVGRG   158
sp|P06224|SIGA_BACSU      PVRMYLKEIGRVNLLSAKEEIAYAQKIEEG---DEESKRRLAEANLRLVVSIAKRYVGRG   157
                          **********   *::  **   *  :  *      ::  **************** ***

sp|P33656|SIGA_CLOAB      MLFLDLIQEGNLGLIKAVEKFDFKKGFKFSTYATWWIRQAITRAIADQARTIRIPVHMVE   224
sp|Q04506|SIGA_LACLA      MQFLDLIQEGNMGLMKAVDKFDHTKGFKFSTYATWWIRQAITRAIADQARTIRIPVHMVE   233
sp|P58290|SIGA_LACLC      MQFLDLIQEGNMGLMKAVDKFDQTKGFKFSTYATWWIRQAITRAIADQARTIRIPVHMVE   211
sp|O33662|SIGA_STRMU      MQFLDLIQEGNMGLMKAVDKFDYSKGFKFSTYATWWIRQAITRAIADQARTIRIPVHMVE   216
sp|P0A4J0|SIGA_STRR6      MQFLDLIQEGNMGLMKAVDKFDYSKGFKFSTYATWWIRQAITRAIADQARTIRIPVHMVE   214
sp|P52329|SIGA_ENTFA      MQFLDLIQEGNMGLMKAVEKFDYRKGFKFSTYATWWIRQAITRAIADQARTIRIPVHMVE   214
sp|Q8CP24|SIGA_STAES      MLFLDLIQEGNMGLIKAVEKFDFSKGFKFSTYATWWIRQAITRAIADQARTIRIPVHMVE   214
sp|Q99TT5|SIGA_STAAN      MLFLDLIQEGNMGLIKAVEKFDFNKGFKFSTYATWWIRQAITRAIADQARTIRIPVHMVE   214
sp|Q92BQ6|SIGA_LISIN      MLFLDLIQEGNMGLMKAVEKFDFNKGFKFSTYATWWIRQAITRAIADQARTIRIPVHMVE   220
sp|P52331|SIGA_LISMO      MLFLDLIQEGNMGLMKAVEKFDFNKGFKFSTYATWWIRQAITRAIADQARTIRIPVHMVE   220
sp|O66381|SIGA_BACHD      MLFLDLIQEGNMGLIKAVEKFDYNKGFKFSTYATWWIRQAITRAIADQARTIRIPVHMVE   218
sp|P06224|SIGA_BACSU      MLFLDLIQEGNMGLMKAVEKFDYRKGYKFSTYATWWIRQAITRAIADQARTIRIPVHMVE   217
                          * *********:**:***:***  **:*********************************

sp|P33656|SIGA_CLOAB      TINKLIRVSRQLLQELGREPQPEEIAKIMDMPVDKVREIMKIAQEPVSLETPIGEEEDSH   284
sp|Q04506|SIGA_LACLA      TINKLIRVQRNLLQELGRDPSPEEIGKELHMAPDKVREVLKIAQEPVSLETPIGEEDDSH   293
sp|P58290|SIGA_LACLC      TINKLIRVQRNLLQELGRDPSPEEIGKELHMAPDKVREVLKIAQEPVSLETPIGEEDDSH   271
sp|O33662|SIGA_STRMU      TINKLVREQRNLLQELGQDPTPEQIAERMDMTPDKVREILKIAQEPVSLETPIGEEDDSH   276
sp|P0A4J0|SIGA_STRR6      TINKLVREQRNLLQELGQDPTPEQIAERMDMTPDKVREILKIAQEPVSLETPIGEEDDSH   274
sp|P52329|SIGA_ENTFA      TINKLIRIQRQLLQDLGREPTPEEIGAEMDLPTEKVREILKIAQEPVSLETPIGEEDDSH   274
sp|Q8CP24|SIGA_STAES      TINKLIRVQRQLLQDLGRDPAPEEIGEEMDLPPEKVREILKIAQEPVSLETPIGEEDDSH   274
sp|Q99TT5|SIGA_STAAN      TINKLIRVQRQLLQDLGRDPAPEEIGEEMDLPAEKVREVLKIAQEPVSLETPIGEEDDSH   274
sp|Q92BQ6|SIGA_LISIN      TINKLIRVQRSLLQDLGRDPSPEEIGEEMDLPTEKVREILKIAQEPVSLETPIGEEDDSH   280
sp|P52331|SIGA_LISMO      TINKLIRVQRSLLQDLGRDPSPEEIGEEMDLPTEKVREILKIAQEPVSLETPIGEEDDSH   280
sp|O66381|SIGA_BACHD      TINKLIRVQRQLLQDLGREPSPEEVAEEMDLTPEKVREILKIAQEPVSLETPIGEEDDSH   278
sp|P06224|SIGA_BACSU      TINKLIRVQRQLLQDLGREPTPEEIAEDMDLTPEKVREILKIAQEPVSLETPIGEEDDSH   277
                          *****:* .*.***:**::* **::.  :.:  :****::****************:***

sp|P33656|SIGA_CLOAB      LGDFIPDDEAPAPADAAAFRMLKEQLLKILNTLTPREEKVLRLRFGLDDGRARTLEEVGK   344
sp|Q04506|SIGA_LACLA      LGDFIEDDVIESPVDYTNRILLREQLDEVMDTLTDREENVLRMRFGLDDGRMHTLEDVGK   353
sp|P58290|SIGA_LACLC      LGDFIEDDVIESPVDYTNRILLREQLDEVMDTLTDREENVLRMRFGLDDGRMHTLEDVGK   331
sp|O33662|SIGA_STRMU      LGDFIEDEVIENPVDYTTRVVLREQLDEVLDTLTDREENVLRLRFGLDDGKMRTLEDVGK   336
sp|P0A4J0|SIGA_STRR6      LGDFIEDEVIENPVDYTTRIVLREQLDEILDTLTDREENVLRLRFGLDDGKMRTLEDVGK   334
sp|P52329|SIGA_ENTFA      LGDFIEDQDATSPAEHAAYELLKEQLEDVLDTLTDREENVLRLRFGLDDGRTRTLEEVGK   334
sp|Q8CP24|SIGA_STAES      LGDFIEDQEAQSPSDHAAYELLKEQLEDVLDTLTDREENVLRLRFGLDDGRTRTLEEVGK   334
sp|Q99TT5|SIGA_STAAN      LGDFIEDQEAQSPSDHAAYELLKEQLEDVLDTLTDREENVLRLRFGLDDGRTRTLEEVGK   334
sp|Q92BQ6|SIGA_LISIN      LGDFIEDQDATSPSDHAAYELLKEQLEDVLDTLTDREENVLRLRFGLDDGRTRTLEEVGR   340
sp|P52331|SIGA_LISMO      LGDFIEDQDATSPSDHAAYELLKEQLEDVLDTLTDREENVLRLRFGLDDGRTRTLEEVGR   340
sp|O66381|SIGA_BACHD      LGDFIEDQDALAPSDAAAYELLKEQLEDVLDTLTDREENVLRLRFGLDDGRTRTLEEVGK   338
sp|P06224|SIGA_BACSU      LGDFIEDQEATSPSDHAAYELLKEQLEDVLDTLTDREENVLRLRFGLDDGRTRTLEEVGK   337
                          ***** *:    * : :   :*:*** .:::*** ***:***:*******: :***:**:

sp|P33656|SIGA_CLOAB      EFNVTRERIRQIEAKALRKLRHPSRSKKLKDYLD-   378
sp|Q04506|SIGA_LACLA      QFKVTRERIRQIEAKAIKKLRHPRRSKPLRDFM--   386
sp|P58290|SIGA_LACLC      QFKVTRERIRQIEAKAIKKLRHPRRSKPLRDFM--   364
sp|O33662|SIGA_STRMU      VFDVTRERIRQIEAKALRKLRHPSRSKQLRDFVED   371
sp|P0A4J0|SIGA_STRR6      VFNVTRERIRQIEAKALRKLRQPSRSKPLRDFIED   369
sp|P52329|SIGA_ENTFA      VFGVTRERIRQIEAKALRKLRHPSRSKQLKDFLE-   368
sp|Q8CP24|SIGA_STAES      VFGVTRERIRQIEAKALRKLRHPSRSKRLKDFMD-   368
sp|Q99TT5|SIGA_STAAN      VFGVTRERIRQIEAKALRKLRHPSRSKRLKDFMD-   368
sp|Q92BQ6|SIGA_LISIN      VFGVTRERIRQIEAKALRKLRHPSRSKQLKDFLE-   374
sp|P52331|SIGA_LISMO      VFGVTRERIRQIEAKALRKLRHPSRSKQLKDFLE-   374
sp|O66381|SIGA_BACHD      VFGVTRERIRQIEAKALRKLRHPSRSKRLKDFLE-   372
sp|P06224|SIGA_BACSU      VFGVTRERIRQIEAKALRKLRHPSRSKRLKDFLE-   371
                           * *************::***:* *** *:*::
```

Figure S5. **Sequence alignment of the RNA polymerase sigma factor SigA.** The conserved DLIQ motif (boxed in black), which is involved in the interaction with polymerase core subunit RpoC, and the helix-turn-helix (HTH) DNA-binding motif (boxed in grey) are indicated. Reference strains are (see Supplementary Table S3): *Lactococcus lactis* subsp. lactis (strain IL1403), *Clostridium acetobutylicum* (strain DSM 792), *Listeria innocua* serovar 6a (strain CLIP 11262), *Listeria monocytogenes* serovar 1/2a (strain EGD-e), *Bacillus halodurans* (strain C-125), *Bacillus subtilis* (strain 168), *Streptococcus mutans* serotype c (strain UA159), *Streptococcus pneumoniae* (strain R6), *Staphylococcus epidermidis* (strain FDA PCI 1200), *Staphylococcus aureus* (strain N315), *Enterococcus faecalis* (strain V583), *Lactococcus lactis* subsp. cremoris (strain ATCC 19257). Asterisks (*) indicate positions that have a fully conserved residue, while colons (:) or dots (.) indicate conservation between groups of strongly or weakly similar properties, respectively. Colour codes: red, small amino acids; blue, acidic residues; and magenta, basic residues. The rest of the amino acids (containing hydroxyl, sulfhydryl or amine groups) are indicated in green.

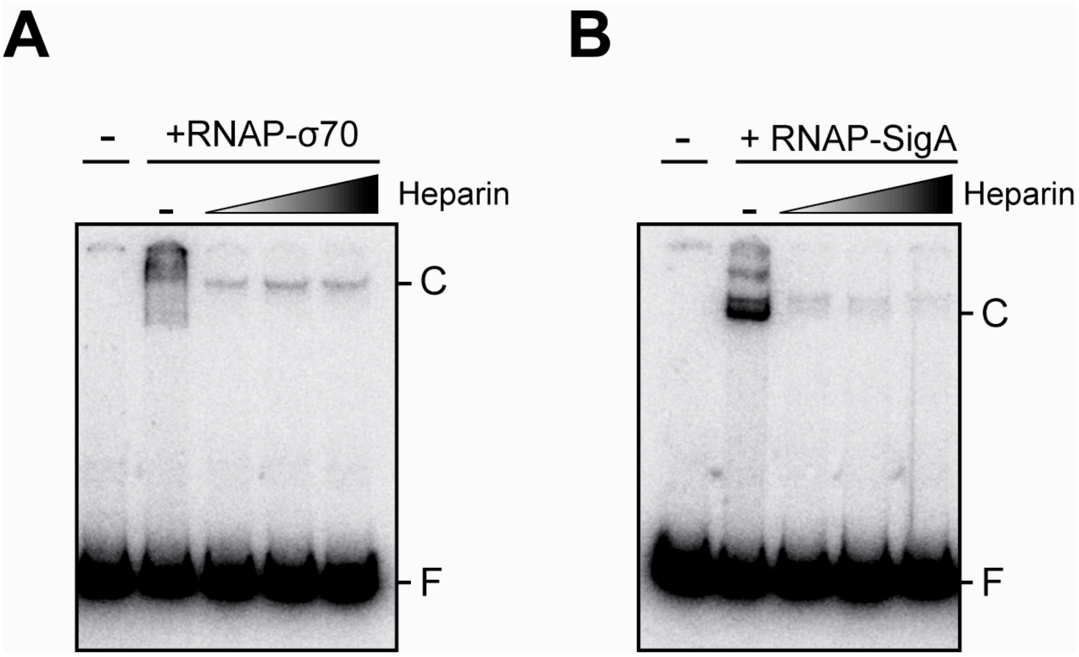

Figure S6. Interaction of RNAP-σ70 and RNAP-SigA with a 222-bp DNA fragment that contains the *P1623B* and *Pmga* divergent promoters. Electrophoretic mobility shift assays (EMSA) were performed using a radioactively labelled 222-bp DNA fragment (coordinates 1598519-1598298 of the pneumococcal R6 genome). This fragment contains the *P1623B* and *Pmga* divergent promoters (see Solano-Collado et al., 2013). Oligonucleotides 1622H (5′-CGGATTAAACCTCTTGCAATTATACC-3′) and 1622I (5′-CAAATTCTTTAATTGTTGCTATTA-3′) were used for PCR amplification of the 222-bp DNA region, as previously described (Solano-Collado et al., 2013). Radioactive labelling of the 222-bp DNA fragment was carried out as reported (Solano-Collado et al., 2013). EMSA experiments were performed with 2 nM of ^{32}P-labelled DNA using **(A)** the *E. coli* RNA polymerase holoenzyme that contains the σ70 factor (RNAP-σ70) (0.5 units; Epicentre), and **(B)** the holoenzyme constituted by the *E. coli* RNA polymerase core and the SigA factor (RNAP-SigA) (see Materials and Methods; *in vitro* transcription assays: *Reconstitution step*). Binding reactions were incubated at 30ºC for 15 min. Then, heparin was added at different concentrations (10, 20 and 40 μg/ml) and reactions were incubated for a further 5 min. Reaction mixtures were loaded onto a native gel (5% polyacrylamide). Bands corresponding to unbound DNA (F) and RNA polymerase-DNA complexes (C) were visualized using a Fujifilm Image Analyzer FLA-3000. RNA polymerase-DNA complexes with much slower electrophoretic mobility than the free DNA were detected. Such complexes were unstable to heparin challenge.

Reference

Solano-Collado, V., Lurz, R., Espinosa, M., and Bravo A. (2013). The pneumococcal Mga*Spn* virulence transcriptional regulator generates multimeric complexes on linear double-stranded DNA. *Nucleic Acids Res.* 41, 6975-6991. doi: 10.1093/nar/gkt445

Table S1

List of 36 representative *Streptococcus* strains selected after performing a Megablast by using the *sigA* (*rpoD*) gene sequence of *S. pneumoniae* R6 as query

***Streptococcus* species**	**Human pathogen**	**Genome reference**	**Query Cover**	**Per. ident**	**Alignment length**	**Mismatches**	**Gap opens**	**Query start**	**Query end**	**Subject start**	**Subject end**
*S. pneumoniae R6**	Yes	AE007317.1	100%	100.00	1110	0	0	965281	966390	965281	966390
S. mitis SVGS_061	Yes	CP014326.1	100%	94.59	1110	60	0	965281	966390	799762	800871
S. gwangjuense ChDC B345	Yes	CP032621.1	100%	93.87	1110	68	0	965281	966390	694363	695472
S. pseudopneumoniae IS7493	Yes	CP002925.1	99%	93.06	1109	77	0	965281	966389	990381	991489
S. oralis subsp. dentisani F0392	Yes	CP034442.1	100%	88.47	1110	128	0	965281	966390	685616	684507
S. australis NCTC5338	Yes	LR594040.1	99%	87.83	1109	135	0	965281	966389	1145683	1144575
S. parasanguinis FW213	Yes	CP003122.1	100%	82.82	1112	187	4	965281	966390	798448	799557
S. suis CZ130302	Yes	CP024974.1	98%	82.55	1100	186	4	965290	966386	1134239	1135335
S. viridans NCTC3166	Yes	LR134266.1	100%	82.09	1117	189	6	965281	966390	716686	717798
S. koreensis JS71	Yes	CP032620.1	100%	81.99	1116	192	5	965281	966390	1845344	1844232
S. gordonii NCTC3165	Yes	LS483375.1	100%	81.90	1116	193	5	965281	966390	1260558	1259446
S. intermedius TYG1620	Yes	AP014880.1	100%	81.13	1113	207	1	965281	966390	982074	983186
S. cristatus NCTC12479	Yes	LS483383.1	100%	81.27	1116	200	6	965281	966390	1001556	1000444
S. constellatus subsp. pharyngis C1050	Yes	CP003859.1	99%	80.83	1111	209	2	965281	966389	919510	920618
S. anginosus J4206	Yes	CP012719.1	99%	80.67	1112	212	1	965281	966389	929051	930162
S. milleri strain NCTC10708	Yes	LR134307.1	99%	80.81	1115	205	5	965281	966389	851596	852707
S. periodonticum KCOM 2412	Yes	CP034543.1	99%	80.49	1112	214	1	965281	966389	869884	870995
S. canis NCTC12191	Yes	LR134293.1	99%	78.62	1118	214	8	965287	966390	1862635	1861529

S. dysgalactiae subsp. equisimilis NCTC6181	Yes	LR134316.1	99%	78.72	1109	229	5	965287	966390	2024324	2023218
S. infantarius subsp. infantarius CJ18	Yes	CP003295.1	99%	78.45	1109	230	3	965288	966390	1222601	1221496
S. urinalis NCTC13766	Yes	LR134323.1	99%	78.60	1112	223	9	965288	966390	1314770	1313665
S. lutetiensis NCTC8796	Yes	LR594044.1	99%	77.93	1110	234	5	965288	966390	677024	678129
S. acidominimus NCTC11291	Yes	LT906454.1	100%	77.89	1117	236	7	965281	966390	839562	840674
S. gallolyticus subsp. gallolyticus ATCC43143	Yes	AP012053.1	99%	77.53	1108	242	4	965288	966390	1422923	1421818
S. pluranimalium TH11417	Yes	CP025536.1	100%	77.27	1122	234	7	965281	966390	628164	629276
S. mutans B04Sm5	Yes	CP061071.1	97%	78.06	1094	221	11	965308	966390	1231913	1230828
S. uberis NZ01	Yes	CP022435.1	99%	77.30	1110	241	6	965288	966390	685159	686264
S. pyogenes ATCC 19615	Yes	CP008926.1	99%	77.06	1116	235	7	965287	966390	1603659	1604765
S. sobrinus NCTC10921	Yes	LS483381.1	99%	77.24	1116	238	9	965281	966388	707155	708262
S. equi subsp. zooepidemicus NCTC12090	Yes	LS483328.1	99%	76.76	1110	247	6	965288	966390	925849	926954
S. agalactiae ILRI005	Yes	HF952105.1	98%	76.91	1100	242	8	965294	966386	1429653	1428559
S. salivarius ICDC2	No, commensal	CP018187.1	99%	79.25	1113	214	6	965288	966390	1716997	1715892
S. vestibularis NCTC12167	No, commensal	LR134275.1	99%	78.87	1112	220	4	965288	966390	1448562	1447457
S. ratti ATCC 31377	No, commensal	CP043405.1	97%	76.58	1093	239	5	965308	966390	1541584	1542669
S. thermophilus N4L	No, probiotic	LS974444.1	98%	79.96	1103	212	3	965294	966390	50208	51307
S. macedonicus ACA-DC 198	No, probiotic	HE613569.1	98%	76.85	1106	239	4	965295	966390	1291683	1290585

*Sequence of the *sigA* (*rpoD*) gene (locus tag: SPR_RS04905) from *S. pneumoniae* R6 strain was used as query for performing a Megablast (https://blast.ncbi.nlm.nih.gov/Blast.cgi; date: October 24th, 2020). Only one representative strain of those species related to human (pathogenic, commensal or probiotic) was selected from retrieved sequences.

Table S2

Sequence conservation between the pneumococcal SigA factor (*Sp*-SigA, 369 residues) and the housekeeping sigma factors SigA from *B. subtilis* (*Bs*-SigA, 371 residues) and σ70 from *E. coli* (*Ec*-σ70, 613 residues; the non-conserved region σNCR_128-372 was omitted in the analysis). Protein sequences were compared using UniProt *Align* tool, which uses Clustal Omega (The UniProt, 2019; Sievers and Higgins, 2014).

	Sp-SigA and *Bs*-SigA			*Sp*-SigA and *Ec*-σ70		
	Nt	Core	FL	Nt	Core	FL
Identity	23%	80%	65%	22%	66%	54%
Identity + Similarity	64%	92%	84%	60%	88%	80%

Nt: N-terminal less conserved region including domain 1.1 (residues 1-96 of *Sp*-SigA, 1-99 of *Bs*-SigA, and 1-95 of *Ec*-σ70).
Core: highly conserved core region consisting of domains 2, 3, and 4 (residues 97-369 of *Sp*-SigA 100-371 of *Bs*-SigA, and 96-613 of *Ec*-σ70 lacking the non-conserved region σNCR_128-372).
FL: Full-length protein.

References

The UniProt Consortium. (2019). UniProt: a worldwide hub of protein knowledge. *Nucleic Acids Res.* 47, D506-D515. doi: 10.1093/nar/gky1049

Sievers, F., and Higgins, D. G. (2014). Clustal Omega, accurate alignment of very large numbers of sequences. In: Russell, D. (eds). Multiple Sequence Alignment Methods. Methods in Molecular Biology (Methods and Protocols). Humana Press, Totowa, N. J., vol 1079, pp. 105-116. doi: 10.1007/978-1-62703-646-7_6

Table S3

Proteins related to SigA from *S. pneumoniae* R6

Entry[a]	Organism	Length	Identity (%)[b]
P0A4J0	*Streptococcus pneumoniae* (strain ATCC BAA-255 / R6)	369	100
O33662	*Streptococcus mutans* serotype c (strain ATCC 700610 / UA159)	371	86.99
P58290	*Lactococcus lactis* subsp. cremoris (Streptococcus cremoris)	364	72.35
Q04506	*Lactococcus lactis* subsp. lactis (strain IL1403) (Streptococcus lactis)	386	70.84
P52329	*Enterococcus faecalis* (strain ATCC 700802 / V583)	368	70.49
Q92BQ6	*Listeria innocua* serovar 6a (strain ATCC BAA-680 / CLIP 11262)	374	67.03
P52331	*Listeria monocytogenes* serovar 1/2a (strain ATCC BAA-679 / EGD-e)	374	67.03
O66381	*Bacillus halodurans* (strain ATCC BAA-125 / DSM 18197 / FERM 7344 / JCM 9153 / C-125)	372	66.58
Q8CP24	*Staphylococcus epidermidis* (strain ATCC 12228 / FDA PCI 1200)	368	65.46
Q99TT5	*Staphylococcus aureus* (strain N315)	368	65.18
P06224	*Bacillus subtilis* (strain 168)	371	64.03
P33656	*Clostridium acetobutylicum* (strain ATCC 824 / DSM 792 / JCM 1419 / LMG 5710 / VKM B-1787)	378	63.31

[a]Identification entry in the UniProtKB (https://www.uniprot.org/).

[b]Identities were obtained from CLUSTAL O (1.2.4) multiple sequence alignments.
